# A four-questions perspective on public information use in sticklebacks (Gasterosteidae)

**DOI:** 10.1098/rsos.181735

**Published:** 2019-02-20

**Authors:** Mike M. Webster, Laura Chouinard-Thuly, Gabor Herczeg, Jun Kitano, Riva Riley, Sean Rogers, Michael D. Shapiro, Takahito Shikano, Kevin N. Laland

**Affiliations:** 1 Centre for Biological Diversity, School of Biology, University of St Andrews, St Andrews, Fife KY16 9TF, UK; 2 Department of Biology, McGill University, 1205 Docteur Penfield, Montréal, Quebec, Canada H3A 1B1; 3 Ecological Genetics Research Group, Department of Biosciences, University of Helsinki, Finland; 4 Behavioural Ecology Group, Department of Systematic Zoology and Ecology, Eötvös Loránd University, Hungary; 5 Division of Ecological Genetics, National Institute of Genetics, Mishima, Japan; 6 Department of Zoology, University of Cambridge, UK; 7 Ecology and Evolutionary Biology, Calgary, Canada; 8 Department of Biology, University of Utah, Salt Lake City, UT 84112, USA

**Keywords:** social learning, social information, foraging, cognition

## Abstract

Whether learning primarily reflects general processes or species-specific challenges is a long-standing matter of dispute. Here, we present a comprehensive analysis of public information use (PI-use) in sticklebacks (Gasterosteidae). PI-use is a form of social learning by which animals are able to assess the relative quality of resources, here prey patches, by observing the behaviour of others. PI-use was highly specific with only *Pungitius* and their closest relative *Culaea inconstans* showing evidence of PI-use. We saw no effects of ontogenetic experience upon PI-use in *Pungitius pungitius*. Experiments with live demonstrators and animated fish revealed that heightened activity and feeding strikes by foraging conspecifics are important cues in the transmission of PI. Finally, PI-use was the only form of learning in which *P. pungitius* and another stickleback, *Gasterosteus aculeatus* differed. PI-use in sticklebacks is species-specific and may represent an ‘ecological specialization’ for social foraging. Whether this reflects selection on perception, attentional or cognitive processes remains to be determined.

## 1. Introduction

The extent to which the learning abilities of animals are shaped by natural selection in response to species-specific ecological challenges or by general processes varying little across taxa has long been a matter of dispute [1–4]. This issue lay at the heart of debates between ethologists and comparative psychologists in mid-twentieth century and has resurfaced in recent discussions of evolutionary psychology, cognitive ecology and cultural evolution [1–10]. Rats (*Rattus norvegicus*) learn to associate sickness with taste more readily than audio-visual cues [6], juvenile songbirds preferentially learn conspecific song [5,7] and some scatter-hoarding songbirds and rodents possess enhanced spatial memory [8,9]. To many, such experiments suggest that learning and memory evolves in response to species-specific ecological demands [10], yet interpretation of these findings remains contested [2]. A comprehensive understanding of how natural selection shapes animal cognition requires analysis of the evolution, ontogeny, mechanism and function of traits, that is, all of Tinbergen's four questions [11].

Social learning in animals is subject to current interest from several academic fields [12–16]. Many researchers have argued that specializations in social learning (including pedagogical cueing, shared attention, ‘motherese’) are critical to human culture and cognition, and underlie the ecological and demographic success of our species [17–19]. Knowledge of how selection has tailored social learning would be facilitated by the identification of specializations among animal models that could be subject to intensive experimental investigation. Evidence for adaptive specialization is found in monkeys that acquire a fear of snakes from conspecifics [20], but, while this has been investigated at behavioural and physiological levels [21], ethical and practical barriers impede detailed exploration of the genetic and neural bases of this capability in primates. As a consequence, the existence of social learning adaptations remains contentious [2,4,22,23].

In this paper, we present a comprehensive analysis of a putative specialization in learning, ‘public information use’ (henceforth PI-use), by species of stickleback fishes (Gasterosteidae). Public information refers to socially transmitted information through which an ‘observer’ is able to estimate the relative quality of two of more resources by monitoring the success of other individuals, termed here ‘demonstrators’, as they exploit them. PI-use is a form of social learning. Here, we focused upon PI-use in a foraging context. Previous work has implied differences in the learning of two species of sticklebacks, with ninespine (*Pungitius pungitius*) but seemingly not threespine (*Gasterosteus aculeatus*) sticklebacks exhibiting PI-use when selecting between rich and poor prey patches [24,25]. In these experiments, *P. pungitius* was more likely to visit and spend time at the rich patch, even after the demonstrators had been removed, while *G. aculeatus*, when subjected to the same test, swim with equal frequency to the rich and poor patches. As the observer's experience was restricted to the relative success of the demonstrators at either prey patch, and because alternative explanations such as residual chemical cues from prey or differences in the salience of the social information provided by the demonstrators of the two species could be ruled out, it was concluded that *P. pungitius* were using PI to select between the patches [24]. *Pungitius* and *Gasterosteus* often form mixed-species shoals [26], are sympatric over extensive freshwater and coastal regions of the temperate Northern Hemisphere, and possess similar life histories [27]. It is therefore germane to enquire why one species should possess a learning capability that the other apparently lacks.

Here, we address this question, employing a *Four Questions* [11] perspective to investigate its phylogenetic distribution (evolution), ontogeny, mechanism and function. First, we asked how PI-use was distributed among stickleback species, comparing PI-use in multiple populations of *Gasterosteus* sp., *Pungitius* sp. and *Culaea inconstans* sampled from throughout their natural ranges. We also collected data on PI-use from single populations of two further stickleback species, *Apeltes quadracus* and *Spinachia spinachia*, both of which have more restricted distributions. In an attempt to identify the effects of local ecology (principally predation pressure) upon PI-use, we also collected data on PI-use in *G. aculeatus* and *P. pungitius* collected from multiple high- and low-predation channels in the UK. Next, we investigated the role of ontogenetic experience in shaping PI-use in *P. pungitius*, the species in which PI-use was most consistently seen. We performed two experiments in which the rearing environments of juvenile fish were manipulated, allowing us to vary the predictability of prey provisioning (in turn affecting the value of using social information) in the first experiment and the social environment, with fish raised alone or in groups (affecting exposure to social information) in the second.

In our third set of experiments, we addressed the mechanism behind PI-use. We broadly interpreted mechanism as encompassing both how and what the animal learns. Earlier work has also shown that in *P. pungitius*, PI is learned [28] and that the observers learn the specific location in which they saw others feeding, but that they cannot generalize between locations with similar landmarks [29]. In the current set of experiments, we investigated attentional and motivational processes, focusing upon the cues produced by feeding demonstrators to which the observers responded. We first identified the cues produced by the demonstrators as they fed at different rates. These experiments were performed for both *G. aculeatus* and *P. pungitius*. Once we had identified demonstrator behaviours that varied predictably with feeding behaviour, we isolated these and presented them to the observers, allowing us to determine which aspects of the demonstrators' behaviour the observers attend to and are attracted to. For *P. pungitius*, we explored these further using novel animated conspecifics as demonstrators, allowing us to precisely and consistently manipulate demonstrator behaviour. Finally, we focused upon the function of PI-use, by comparing other forms of social information use and asocial learning (operant and classical conditioning) between *G. aculeatus* and *P. pungitius* in order to determine whether differences in PI-use between the two species reflect a general difference in learning ability or a specialization, possibly related to foraging, in *P. pungitius*.

## 2. Methods and results

### 2.1. General methods: public information use binary choice assay

**Figure 1.**
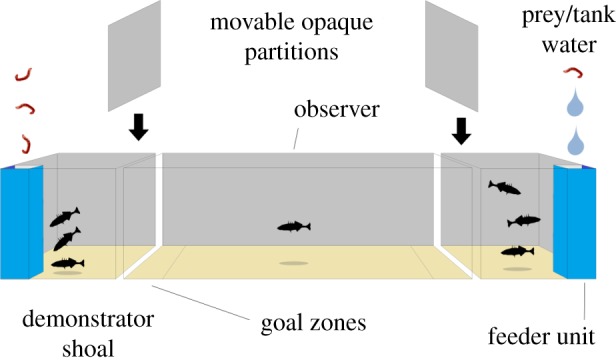
Apparatus and procedure. Plan view of the apparatus used in the PI-use assays. Each trial had three phases. (i) Settling phase, in which three ‘demonstrator’ fish were added to each outer tank and one ‘observer’ fish (the test subject) was added to a removable transparent holding unit in the main central tank. (ii) Demonstration phase, in which food items or ‘blanks’ of tank water were added to the two feeder units, so that one feeder yielded prey at three times the rate of the other, simulating richer and poorer patches. The front of each feeder was transparent, allowing the demonstrators to attack the food as it sank, while the sides were opaque, preventing the focal observer from seeing the food. (iii) Test phase, in which opaque barriers were slid between outer and main tanks, preventing the observer from seeing the demonstrators and, after a short pause, the observer was released from the holding unit. The amount of time the observer spent in each goal zone was recorded as a measure of patch preference.

We used an assay of PI-use in which subjects (termed observers) were able to learn about the relative quality of artificial prey patches attended by two groups of stimulus conspecifics (termed demonstrators), allowing us to test for PI-use under controlled conditions (figure 1). Our general method employed a binary choice test tank comprising a main observer arena, set between two demonstrator chambers, deploying the methods of Coolen *et al*. [24]. Each demonstrator chamber contained three conspecific demonstrators and a feeder unit. The two feeder units released food at different rates, and were carefully designed to allow the observer to see the demonstrators feeding, but not see or otherwise detect the food itself. The observer was therefore able to estimate patch quality only indirectly, by using public information generated by the feeding demonstrators. A goal zone was present at each end of the arena, adjacent to either demonstrator chamber. The amount of time the observer spent in each goal zone was taken as a measure of the observer's preference for the adjacent food patch (i.e. feeder unit). A net preference for the zone adjacent to the rich compared with poor food patch was taken as an indication of PI-mediated patch choice. Observers were tested in trials consisting of three phases: (i) a *settling phase*, in which the demonstrators and observers were allowed to acclimate to the experimental conditions, (ii) a *demonstration phase*, in which the observer was allowed to watch two demonstrator groups feeding at separate locations (and thereby potentially acquire knowledge of the patch quality), and (iii) a *test phase*, in which the demonstrators were hidden from view of the observer, and the observer was released and allowed to choose a food patch (i.e. approach and spend time in the vicinity of the goal zones adjacent to the two demonstrated prey patches). Some experiments used modified versions of this assay, as outlined below.

### 2.2. Set-up

We used a glass tank (45 × 30 × 30 cm, water depth 12 cm) as the observer arena. At either end of the observer arena, we placed colourless Perspex demonstrator chambers (27 × 15 × 12 cm, water depth 12 cm). These were placed 0.5 cm from the ends of the observer chamber. Each of the three tanks contained a 1 cm deep layer of coarse sand. Within the observer arena, yellow plastic bars, 1 cm wide and 1 cm deep, secured to the base of the tank and rising to the surface of the sand divided the tank into three zones. These were set 8 cm from either end of the observer arena. The two areas between the ends of the tank and the bar were designated the prey patch goal zones.

Within each of the demonstrator tanks, we placed a feeder unit. The feeder unit consisted of a 4 × 4 cm base, 30 cm tall tower. The feeder units were placed in the corner of the demonstrator chamber furthest from the observer arena. The front wall of the feeder unit, facing the demonstrators, was transparent, so that the demonstrators could see the prey as it was delivered. The rear wall was white to maximize the visibility of the prey. The side walls were opaque white, so that the observer in the centre of the tank could not see the prey. Demonstrators were unable to reach the prey until it sank to the bottom of the feeder, but were able to attack it as it fell. The front wall of the feeder stopped 1 cm short of the floor of the tank, allowing the demonstrators to eat the prey once it had reached the bottom of the feeder. Prey deliveries consisted of two 3 mm long pieces of thawed frozen bloodworm. These were small enough to be consumed with minimal handling by the demonstrators, ensuring that the observing focal fish could see the feeding behaviour of the demonstrators, but not the prey itself. Screening on the outside of the test tank prevented the fish from seeing the experimenter as the prey were added. (In some trials in the multi-species comparison project described below, *Artemia* or Mysids were used instead of bloodworms. This is because fish were tested in a number of different laboratories, and were fed different standard diets. Within the trials, we used the foods which the fish were used to being fed in order to ensure that the demonstrators were equally familiar with the food across trials. The type of food used in the trials is noted for each population in electronic supplementary material, table S1). Housing the demonstrators in watertight chambers ensured that no chemical cues originating from the prey were available to the observer, as these may provide direct information about feeder location and prey density. This ensured that observer could only base their patch choices upon visual cues received during the demonstration phase.

Within the observer arena, the observer was held within a holding unit for the duration of the settling period and demonstration phase. The holding unit consisted of a tower of clear, colourless perforated Perspex measuring 10 × 10 cm × 15 cm tall. It was attached via a monofilament line to a 15 cm long arm clamped to the top of the observer arena, allowing the holding unit to be raised by the experimenter. The holding unit was placed 5 cm from the side wall of the observer arena and half way between the end walls that abutted the demonstrator chambers.

We used two opaque black plastic screens measuring 30 × 30 cm square by 2 mm thick to separate the observer arena from the demonstrator chambers during the choice phase of the trial. These were designed so that they could be slid into place between the tanks without causing any significant vibration that might alarm the observer. The exterior walls of both the observer arena and demonstrator chambers were screened in black plastic. Observations were made via a webcam fixed 90 cm above the tank and connected to a laptop computer.

### 2.3. Subjects

Specific details on the test subjects (such as species and population of origin) are given in the methods for the specific experiments, described below, and in electronic supplementary material, tables S1 and S2. We only used non-reproductive fish as demonstrators or observers, as reproductive state is known to influence PI-use [30]. Neither demonstrators nor observers were sexed. Within trials, the observers and demonstrators were matched to each other by body length to within 3 mm. Since the demonstrators were typically drawn from a limited pool of available fish, the precise number of which varied between populations, some demonstrators were used in multiple trials. No individual, however, was used more than once in any 3-day period. Observers were only tested once, unless otherwise stated.

### 2.4. Procedure

The demonstrators and focal fish were deprived of food for 24 h before testing in order to ensure that they were motivated to feed. Then three demonstrators were added to each demonstrator chamber and allowed to settle for 10 min before the focal fish was added to the central holding unit and allowed to settle for a further 10 min. The demonstration phase lasted for 6 min and ran as follows. At the beginning of the first, third and fifth minute of the trial, prey suspended in 1 cm^3^ of tank water were added to the feeder in the designated rich patch, using a pipette. During the first and third minutes of the trial, the poor patch received no prey. A ‘blank’ consisting of 1 cm^3^ of tank water was added to the feeder at the same time that the rich feeder received prey. During the 5th minute, the poor feeder also received prey. This ensured that while prey were delivered at a 3 : 1 ratio, the focal fish was unable to select a prey patch simply on the basis of it being the last place it saw fish feeding. Trials in which the demonstrators did not eat all of the prey were abandoned. The location of the rich patch, either to the left or to the right of the observer arena, was randomly selected for each trial, and accounted for in the statistical analyses outlined for each of the experiments described below.

After the 6 min demonstration phase, the opaque black screens were slid into place between the observer arena and the two demonstrator chambers. This took approximately 10 s and did not appear to stress the observer. The observer was allowed to settle for a further 1 min before being released from the holding unit. The observer was released by raising the holding unit 5 cm from the base of the arena, using the pulley mechanism. The base of the holding unit was left suspended beneath the water surface, so as not to disturb the surface of the water and startle the test observer. This commenced the choice phase of the trial, which lasted for 5 min. During the choice phase, unless otherwise stated below, we point sampled the location of the focal fish every 6 s. These data were used in a number of analyses, which are outlined below.

## 3. Part 1: phylogeny: distribution of public information use in sticklebacks

### 3.1. Public information use by species: Gasterosteidae

#### 3.1.1. Methods

Previous research has revealed a species difference in PI-use in sticklebacks; *P. pungitius* were observed to use public information, while *G. aculeatus* were not [24]. Here, we expand on this to include multiple species from the stickleback family, collecting data on PI-use for *Gasterosteus* sp. *n* = 16 populations, 420 individuals; *Pungitius* sp. *n* = 15 populations, 382 individuals; *A. quadracus n* = 1 population, 20 individuals; *S. spinachia n* = 1 population, 19 individuals; *C. inconstans n* = 6 populations, 104 individuals (electronic supplementary material, table S1). Each individual was tested for PI-use once, using the procedure described above. Experiments were carried out in laboratories at the Universities of Calgary (Canada), Helsinki (Finland), Utah (Salt Lake City, USA), St Andrews (UK), the Fred Hutchinson Cancer Research Centre (Seattle, USA) and National Institute for Genetics (Mishima, Japan). Housing conditions were similar between laboratories, with fish kept in population-segregated groups in aquaria with sand or gravel substrate, and filtered and aerated water. All were held at a constant temperature under artificial light. Light : dark cycles varied between 12 : 12 and 16 : 8 between the different facilities. Conspecifics were used as demonstrators. For prey deliveries during the trials, we used the food that the fish were maintained on, and that they were therefore most familiar with, in their home laboratories (electronic supplementary material, table S1). We recorded two response variables: the first goal zone entered by the fish and the difference in time allocation to the two goal zones (time spent in rich goal zone − time spent in poor goal zone).

#### 3.1.2. Statistical analyses

**Figure 2.**
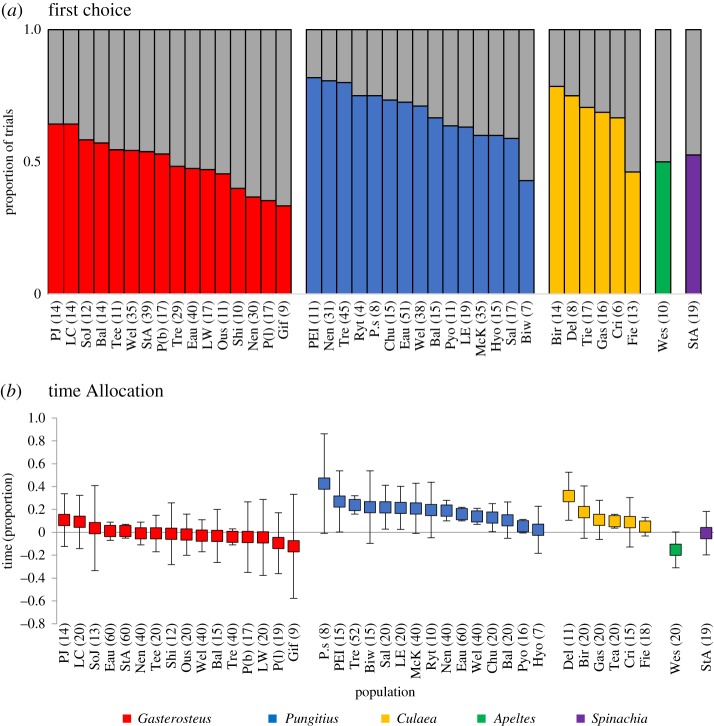
Phylogeny: distribution of PI-use in sticklebacks. (*a*) First goal zone entered. The coloured/grey section of the bar shows the proportion of fish that entered the rich/poor goal zone first. We saw an effect of species, with *Pungitius* and *Culaea* entering the rich patch first more often than *Gasterosteus*. Sample sizes for each population are provided in parentheses following each population label. For further details, see electronic supplementary material, tables S1 and S2. (*b*) Time allocation, the mean proportion of time spent in rich patch − time in poor patch (±95% CI). Further details on the populations are reported in electronic supplementary material, tables S1 and S2. Data were analysed using a GLM, reported in the main text. A Tukey HSD post hoc analysis revealed that *Pungitius* and *Culaea* spent more time closer to the demonstrated rich patch than did *Gasterosteus* or *Apeltes* but that they did not differ from *Spinachia* (*Pungitius* compared with *Culaea*, *Gasterosteus*, *Apeltes* and *Spinachia*, *p* = 0.76, less than 0.001, 0.003, 0.267. *Culaea* compared with *Gasterosteus*, *Apeltes* and *Spinachia*: *p* = 0.015, 0.033, 0.654. *Gasterosteus* compared with *Apeltes* and *Spinachia*: *p* = 0.521, 0.999. *Apeltes* compared with *Spinachia*: *p* = 0.787).

*First choice*. We used the first goal zone entered, rich or poor, as our first measure of PI-use. Data were analysed using binary logistic regression. We omitted data from fish that did not enter either goal zone from the analysis (leaving *n* = 745, see figure 2*a* for sample sizes per population). We included species, population (nested within species), origin (wild-captured or laboratory-bred F1) and the location of the rich patch (left or right) as categorical factors. This analysis was also run without *Apeltes* and *Spinachia*, for which we had only one data on one population each (total *n* = 716).

*Time allocation*. We used the difference in time spent in the two patches (time in rich patch − time in poor patch, ‘time allocation’) as a second measure of PI-use. We included trials where the fish were active but did not enter either goal zone (giving *n* = 945 trials in total). The location of the observer (rich or poor patch goal zones or the central area) was recorded every 6 s, giving a total of 50 point samples per trial. These data were analysed using a GLM with a Poisson distribution. Phylogenetic comparative analyses were not used because they are inappropriate in studies with low numbers of taxa, such as this [31]. Species, population (nested within species), origin (wild-captured or laboratory-bred F1) and the location of the rich patch (left or right) were included as categorical factors. Sample sizes are provided in electronic supplementary material, table S1. We again reran the above analysis, excluding *Apeltes* and *Spinachia*, for which only had one data on one population each.

#### 3.1.3. Results

*First choice*. We saw an effect of species upon first goal zone entered (binary logistic regression, *χ*^2^ = 30.09, d.f. = 4, *p* < 0.001, figure 2*a*), with significant contrasts between *Gasterosteus* and *Pungitius* and *Culaea* (*p* < 0.001 and 0.002) but not *Apeltes* or *Spinachia* (*p* = 0.56 and 0.15). We saw no variation between populations (nested within species) (*χ*^2^ = 1.19, d.f. = 38, *p* = 0.65, nor any effects of origin (*χ*^2^ = 1.64, d.f. = 1, *p* = 0.20), or rich patch location (*χ*^2^ = 0.72, d.f. = 1, *p* = 0.40).

Given that we only had data for a single population of each of *Apeltes* and *Spinachia*, we reran the above analysis excluding these two species, observing the same pattern with respect to the other three species. (Species: *χ*^2^ = 29.75, d.f. = 2, *p* < 0.001 (contrasts between *Gasterosteus* and *Pungitius* and *Culaea*: *p* < 0.001 and 0.04), population (nested within species): *χ*^2^ = 51.99, d.f. = 36, *p* = 0.04, origin: *χ*^2^ = 1.43, d.f. = 1, *p* = 0.23, rich patch location *χ*^2^ = 1.29, d.f. = 1, *p* = 0.26.)

*Time allocation*. We saw differences in PI-use between species, with *Pungitius* and *Culaea* spending more time close to the demonstrated rich prey patches compared with *Gasterosteus*, *Apeltes* and *Spinachia* (figure 2*b*). *Apeltes* exhibited an apparent preference for the demonstrated poor patch, which may also be indicative of PI-use; however, with only one population tested, it is difficult to draw firm conclusions. We saw no evidence of intraspecific variation in PI-use, nor any effect of rich patch location or the origin of the fish (GLM: species: *F*_4,945_ = 9.72, *p* < 0.001, population: (nested within species) = *F*_34,945_ = 0.57, *p* = 0.98, location of rich patch: *F*_1,945_ = 1.22, *p* = 0.27, origin (wild-captured or laboratory F1): *F*_1,945_ = 1.71, *p* = 0.19). For 11 out of 15 *Pungitius* populations and two out of six of the *Culaea* populations, the lower bound of the confidence interval is greater than zero, suggesting a bias towards spending more time in the rich prey patch goal zone. In all of the *Gasterosteus* populations and the single *Spinachia* population, the confidence interval spanned zero, suggesting no preference for either goal zone. *Apeltes* exhibited an apparent preference for the demonstrated poor patch, the upper bound of the confidence interval being less than zero, which may also be indicative of PI-use; however, with only one population tested, it is difficult to draw firm conclusions.

Reanalysing the dataset without *Apeltes* and *Spinachia*, we observed the same pattern with respect to the other three species (GLM: species: *F*_2,906_ = 17.29, *p* < 0.001, population: (nested within species) = *F*_32,906_ = 0.56, *p* = 0.98, location of rich patch: *F*_1,906_ = 1.96, *p* = 0.16, origin: *F*_1,906_ = 1.72, *p* = 0.22).

### 3.2. Public information use: intraspecific variation?

#### 3.2.1. Methods

In this experiment, we investigated the role of predation in shaping PI-use. PI-use might be a risk-averse means of collecting information about the distribution of resources, allowing animals to acquire information without the need for direct sampling, which may expose them to risk [32]. The prevalence of risk-averse behaviours might vary between high- and low-predation environments as a result of factors, including individual experience and learning, through selective mortality, via maternal effects or as a result of natural selection. European minnows (*Phoxinus phoxinus*) were more likely to copy the prey patch choices of others when simulated predation risk was higher, implying flexibility in social information use [33]. In guppies (*Poecilia reticulata*), fish from high-predation populations form more cohesive shoals and engage in collective exploration to a greater extent than do those from low-predation populations [34], and that predation affects the collective motion dynamics of guppy shoals, resulting in more socially responsive interaction rules between group members [35]. Maternal exposure to predation cues has been shown to affect the shoaling behaviour offspring in *G. aculeatus*, resulting in more cohesive shoals, an effect which may be brought about via maternally derived cortisol [36]. Long-term transplant experiments in guppies have demonstrated natural section of anti-predator behaviours too [37].

To identify the relative significance of these factors in potentially shaping PI-use in our study system, it is necessary to compare the behaviour of interest in wild-captured individuals and, to identify or rule out the contribution of experience and maternal effects, respectively, F1 and F2 progeny reared under common conditions [37]. With this in mind, the original aim of this experiment was to identify variation in PI-use and to breed lines of offspring from these. As is reported below, however, we saw no evidence of intraspecific variation in PI-use in either *G. aculeatus* or *P. pungitius*. Consequently, the experiment below presents an analysis of PI-use in wild-captured *G. aculeatus* and *P. pungitius* only from high- and low-predation sites within a number of drainages within the UK. We tested the prediction that fish from high-predation sites would use PI to a greater extent than those from low-predation sites. We analysed a subset of the data presented above in §3.1, focusing upon 280 *G. aculeatus* and 180 *P. pungitius*, from 14 and nine channels situated within seven and four drainage networks, respectively (electronic supplementary material, table S2) using PI-use assay described above. Where it was possible, we compared *G. aculeatus* and *P. pungitius* from the same channels (eight channels), as well as six further locations where only *G. aculeatus* was found, and one where only *P. pungitius* was collected.

Predation risk was quantified for each channel as a binary variable, lower or higher, on the basis of the presence or absence of major fish predators (electronic supplementary material, table S2). This convention follows that adopted by early evolutionary ecologists studying the Trinidadian guppy (*Poecilia reticulata*) system (reviewed by Magurran [38]). There are of course limitations to this approach. First, it does not take into account the magnitude of predation pressure from these predators or from different predator species or size classes. Second, it ignores predation from bird and insect predators, which can be highly mobile and may well prey upon sticklebacks at all locations. Third, our approach does not take into account properties of the physical environment that may influence predator–prey interactions, such as water turbidity, the presence of aquatic vegetation and a structural complexity of the substrate. However, given a lack of long-term data on these parameters in these channels and uncertainty over how well short-term continuous measures of these variables can capture longer-term selection pressures, we argue that the binary classification of channels into lower and higher predation environments is a reasonable compromise. Of the nine channels from which we tested *P. pungitius*, four were classed as lower predation and five as higher predation. For the *G. aculeatus*, five were lower and nine were higher predation.

Around 60 fish of each species (where found) were collected using dip nets from each channel. They were held at the University of St Andrews in single-species, single population groups of 20 in 45 l aquaria at 8°C. Holding aquaria contained a coarse sand substrate, and internal filter and artificial plants for cover. The fish were fed frozen bloodworms once per day unless being tested. They were held under these conditions for several months before being tested. They were tested for PI-use using the binary choice assay described above. For each species, 20 fish from each channel were tested. We focused on first goal zone entered and time allocation to the rich goal zone as our key measure of PI-use.

#### 3.2.2. Statistical analyses

*First choice*. We used the first goal zone entered, rich or poor, as our first measure of PI-use. Data were analysed using binary logistic regression. We omitted data from fish that did not enter either goal zone from the analysis (leaving 361 fish). We included species, predation level (nested with drainage) and the location of the rich patch (left or right) as categorical factors.

*Time allocation*. We used the time in rich patch − time in poor patch (time allocation) as a second measure of PI-use, as described above. These data were analysed using a GLM with a Poisson distribution, with species, predation level (nested with drainage) and the location of the rich patch (left or right) as fixed factors. We included trials where the fish were active but did not enter either goal zone (giving *n* = 460 trials in total, see electronic supplementary material, table S2 for further details).

#### 3.2.3. Results

**Figure 3.**
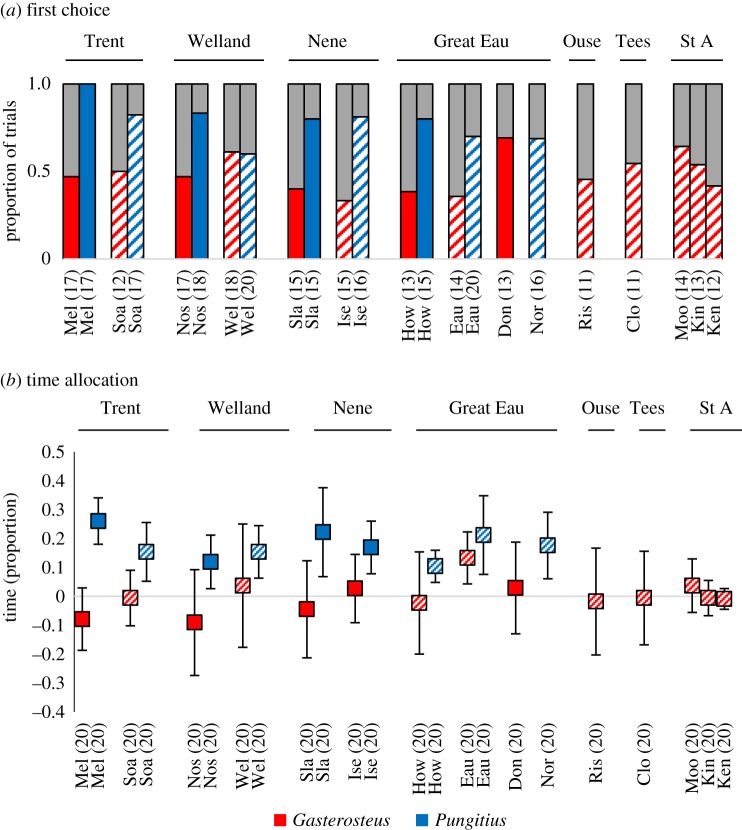
PI-use: Intraspecific variation? (*a*) First goal zone entered. The coloured/grey section of the bar shows the proportion of fish that entered the rich/poor goal zone first. (*b*) Time allocation (mean proportion of time spent in rich patch − time in poor patch, ±95% CI). In each panel, the labels above each set of bars or points identify the drainage and the labels below refer to the separate channels within each drainage. Solid and hatched bars indicate low- and high-predation channels. Sample sizes for each population are provided in parentheses following each channel label. For further details, see electronic supplementary material, table S2. In both cases, we saw a species difference, with *Pungitius* being more likely to enter the rich patch first (*a*) and to spend more time there (*b*) than *Gasterosteus*, with no effect of predation apparent.

*First choice*. *Pungitius* were more likely to enter the rich goal zone first than were *Gasterosteus* (binary logistic regression, *χ*^2^ = 26.62, d.f. = 1, *p* < 0.001, figure 3*a*), with no effects of predation pressure (*χ*^2^ = 1.18, d.f. = 1, *p* = 0.88) or location of the rich patch (*χ*^2^ = 0.14, d.f. = 1, *p* = 0.71) apparent.

*Time allocation*. We saw a clear species difference in PI-use with *Pungitius* spending more time closer to the demonstrated rich patch than *Gasterosteus* (GLM: species: *F*_1,460_ = 37.08, *p* < 0.001, figure 3*b*). We saw no effects of either predation (*F*_1,460_ = 0.32, *p* = 0.97) or location of the rich patch (*F*_1,460_ = 1.85, *p* = 0.17). In all but one of the *Gasterosteus* samples presented in figure 3*b*, the confidence intervals spanned zero, suggesting no preference for the rich or poor prey patch goal zones. For all nine of the *Pungitius* samples, the lower bound of the confidence interval is greater than zero, suggesting a bias towards spending more time in the rich prey patch goal zone.

## 4. Part 2: ontogeny: how is public information use affected by rearing environment?

### 4.1. Part 2: methods

Our experiments in part 1 revealed an apparently robust species-level difference in PI-use. Furthermore, we saw no differences in PI-use between wild-captured and laboratory-reared *P. pungitius* and *G. aculeatus*. Focusing just on *P. pungitius*, in this section, we experimentally investigated the effect of rearing environment upon PI-use, first comparing fish that had been reared under conditions where prey distribution was predictable or unpredictable, and second comparing fish that were raised alone or in groups. In these experiments, we used wild-captured juvenile *P. pungitius*. In June 2008 (prey distribution) and June 2009 (rearing density), we collected several hundred juveniles (less than 9 mm body length) from Melton brook, UK (electronic supplementary material, table S2). These were collected from a stretch measuring several hundred metres long and were allowed to mix in large holding tanks for a day prior to being assigned haphazardly to the rearing treatments.

#### 4.1.1. Prey distribution

In the prey distribution experiment, juvenile fish were reared for five months in tanks in which prey could be delivered at each of four different locations. For each rearing tank, food delivery was either fixed, such that food was delivered in the same place and at the same time, every day, or varied, with food being delivered to a different, randomly selected location at some variable point in time within an 8 h period each day.

Rearing tanks measured 30 × 30 × 30 cm (depth 28 cm) with a 2 cm deep fine sand substrate. Each was screened with black plastic to eliminate outside disturbance. In each corner, we fitted a 2 cm diameter opaque plastic tube running from 10 cm above the water surface to 2 cm above the surface of the substrate. These served as prey delivery tubes, through which prey could be injected using a pipette. The use of tubes prevented the fish from seeing the prey until it arrived just above the bottom of the tank. Each tank contained an air stone, located in the centre. We performed a 30% water change once per week.

We established 40 such tanks in June 2008, and added four fish to each, giving 160 fish in total. The fish measured 5–8 mm standard length, and were drawn haphazardly from a pool of several hundred fish held in a single holding tank. Fish were size matched to within 1 mm within tanks. Half of the tanks were randomly assigned to the fixed prey delivery treatment and half to the varied prey delivery treatment. Fish in the two treatment groups did not differ significantly in their starting size (one-way ANOVA: *F*_1,158_ = 0.11, *p* = 0.60).

In the fixed prey treatment group, the fish were fed at 14.00, via the same food delivery tube. In the varied prey treatment group, the fish received their food ration at a different time each day, at a randomly predetermined hour between 10.00 and 18.00 and via randomly selected tube. All varied treatment tanks received their food at the same time on any given day.

The food, delivered in 4 cm^3^ of tank water, described below, was pipetted vertically down the tube so as to ensure that it did not stick to the sides of the tube. The diet of the fish was changed over the course of the experiment as they grew. From June until August, they were fed 0.5 cm^3^ frozen *Cyclops* sp. per tank per day. These were thawed in 3.5 cm^3^ tank water. From August until October, the fish were fed on 0.5 cm^3^ frozen *Daphnia* per tank per day, and from October until November, they received sixteen 5 mm long sections frozen bloodworms per tank per day.

These treatments ran for five months from mid-June to mid-November, after which the fish were tested for PI-use using the standard PI assay. We lost some fish to mortality over the course of the experiment, and we only tested fish from tanks where three or more individuals remained at the end of the rearing period. In the fixed prey delivery treatment, all 20 tanks had three or four fish at the end of the experiment (five had all four fish and 15 had three fish, with 15 having died over the course of the experiment). In the varied food delivery treatment, two tanks lost all of their fish. Of the remaining 18 tanks, three had all four fish and 15 had three fish remaining (23 having died during the experiment). At test, the fish measured between 28 and 35 mm in length. We saw no body size differences between fish from the two treatments (mean body length per replicate tank, one-way ANOVA: *F*_1,37_ = 0.15, *p* = 0.71).

#### 4.1.2. Rearing density

We established 40 rearing tanks. Twenty of these contained a single fish, and 20 contained four fish. The fish measured 7–9 mm standard length at the time the populations were established, and were drawn haphazardly from a pool of approximately 150 held in a single holding tank. Fish were size matched to within 1 mm within tanks. Fish in the two treatment groups did not differ significantly in their starting size (one-way ANOVA: *F*_1,98_ = 0.06, *p* = 0.81). These treatments ran for five months from mid-June to mid-November, after which the fish were tested for PI-use using the standard PI-use assay. The rearing tanks, laboratory conditions and food types were as described above, with the following exceptions. All tanks were fed at 14.00 each feeding day, via a different prey deliver tube, at a randomly predetermined location each day. The single fish tanks each received one-quarter the prey ration as those with four fish. At the end of the rearing period, five of the single-reared fish had died, leaving 15 fish to be tested. In the group rearing treatments, seven tanks had only two remaining fish. These were excluded. Of the remaining 13 tanks, three had all four fish surviving and 10 had three survivors. A total of 29 fish died during the rearing period. At test, the fish measured between 25 and 33 mm in length. We saw no body size differences between fish from the two treatments (mean body length per replicate tank, one-way ANOVA: *F*_1,27_ = 0.01, *p* = 0.98).

### 4.2. Part 2: statistical analyses

For both experiments, we compared the two treatment groups using a GLMM with treatment included as a fixed effect and rearing tank as a random factor. In the prey distribution (§4.1.1) experiment, the sample sizes were predictable, *n* = 65, and unpredictable, *n* = 57, while in the rearing density (§4.1.2), they were raised alone, *n* = 15 and raised in groups, *n* = 42.

### 4.3. Part 2: results

**Figure 4.**
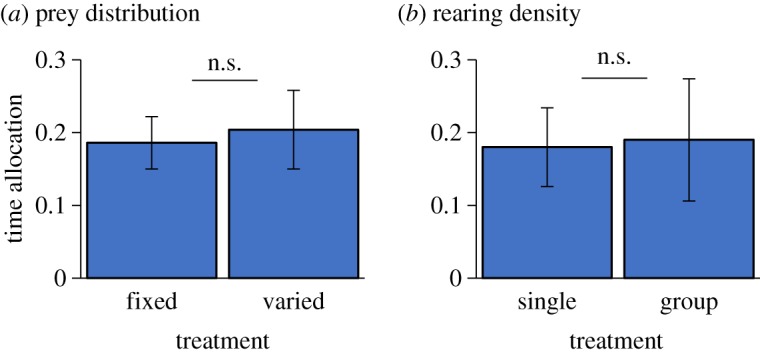
Experimental investigation of the effects or rearing environment upon PI-use in *Pungitius*. Time allocation refers to the mean proportion of time spent in rich patch − time in poor patch, ±95% CI). (*a*) Fish were raised under conditions where prey provision was either fixed or varied and (*b*) fish were raised alone or in groups. In both experiments, there was no significant difference (n.s.) between treatment groups.

We saw no differences in PI-use between the fish in the two rearing treatments in either experiment (prey distribution, GLMM: treatment *χ*^2^ = 0.31, d.f. = 1, *p* = 0.58, rearing tank *χ*^2^ = 7.84, d.f. = 36, *p* = 0.99; rearing density, GLMM: *χ*^2^ = 0.57, d.f. = 1, *p* = 0.81, rearing tank * χ*^2^ = 16.36, d.f. = 26, *p* = 0.18, figure 4). Fish in all four treatment conditions across the two experiments showed evidence of PI-use, indicated by positive time allocation scores and the lower bound of the confidence interval being greater than zero.

Our finding that the development of PI-use is rather inflexible—we were unable to induce changes in PI-use behaviour in our test subjects despite raising them under markedly different conditions—is surprising. These observations may suggest that PI-use develops in *P. pungitius* irrespective of whether individuals have prior opportunities to learn socially, though we have to be cautious in such an interpretation, because we used wild-captured juveniles which may have already acquired the capacity for PI-use prior to being collected. These findings contrast markedly with another study of the development of social learning in this species and in *G. aculeatus*, in which both species readily learned to use social cues to anticipate the presence of food in a novel feeding task after just a few weeks exposure to feeding demonstrators and opportunity to feed [39]. That PI-use apparently develops so rigidly in *P. pungitius* is at odds with recent arguments that social learning has a predominantly associative basis [4], a point we return to in the discussion.

## 5. Mechanism: how is public information transmitted?

In this series of experiments, we investigated the mechanism by which information is transmitted between foraging demonstrators and observers. Earlier work has identified delayed local enhancement, attraction to the specific location where others were earlier seen foraging, to be the process behind it [24,28,29]. Here, we asked: what are the cues that are generated by feeding demonstrators that the observers attend to? We tested both *G. aculeatus* and *P. pungitius*, in order to identify any interspecific differences in demonstrator behaviour that might explain the species differences in PI-use outlined in part 1. Both species were collected from Melton brook, Leicester, UK (electronic supplementary material, table S2). Unsexed, non-reproductive adults measuring 35–40 mm were used. Within trials, all fish were size matched to within 2 mm. Pilot experiments (electronic supplementary material, part S1 and figure S1) identified aspects of demonstrator behaviour that covaried with prey delivery rate. As demonstrator feeding rate increased, the activity and feeding strike rate of the demonstrators also increased, while (in *P. pungitius* only) their distance from the feeder unit and the distance between the individual demonstrators decreased (electronic supplementary material, figure S5). We first isolated these demonstrator behaviours (§5.1) and presented them to observers in order to determine which were involved in the transmission of PI. Next, having found that demonstrator activity and strike rate were both attractive to observers, we presented these cues antagonistically in order to attempt to determine the relative importance of each (§5.2). In a third set of experiments (§5.3), we developed and used computer-animated demonstrators in order to more precisely control and manipulate demonstrator behaviour. This final experiment tested *P. pungitius* only.

### 5.1. Isolating demonstrator feeding cues

#### 5.1.1. Methods

These experiments presented observers with demonstrators engaged in isolated behaviours that were shown to covary with feeding from rich or poor patches (electronic supplementary material, part S1, Pilot experiment and figure S1): more versus less active demonstrators, demonstrator groups performing more or fewer feeding strikes, more or less cohesive demonstrator groups and groups that were closer to or further from the feeder. No food was provided to the demonstrators in these experiments, with the target behaviours being controlled through experimental manipulations. We tested both *G. aculeatus* and *P. pungitius*, using conspecifics as demonstrators. No observer fish was tested more than once. Demonstrators were used multiple times, but no single demonstrator was used more than once in any 72 h period. For each of the four cues, we performed two observer treatments. One was tested in real time, while the demonstrators were still present, while the other was tested after the demonstrators had been removed (simulating PI-use, and testing whether the observers could learn where the demonstrators were previously feeding at a higher rate). In the real-time assay, the observer was allowed to observe the two shoals during a 6 min demonstration phase. Opaque barriers were then placed between the observer and demonstrator chambers for 1 min, as for the standard PI-use binary choice assay, except that here the demonstrators were retained. The barriers were then carefully removed, revealing the demonstrators again, and the observer was released from its holding unit beginning the trial. Adding and then removing the opaque barriers allowed us to control for any effect that this may have had upon the observer's behaviour. We observed no change in the demonstrators' behaviour, and no startle responses, when the barriers were added and removed. In the PI treatment, the observer was allowed to observe the demonstrators for 6 min, as above, after which the opaque barriers were added, the demonstrators were removed and the barriers were taken out again, revealing empty demonstrator tanks. After a 60 s delay, the observer was released, beginning the trial. We performed 20 trials per treatment per species.

*Activity*. Demonstrator activity levels were manipulated by holding the demonstrators in water of differing temperatures (8°C and 18°C). A pilot study (electronic supplementary material, part S2 and figure S2) established that fish held in warmer water were more active than those in cooler water. In the laboratory, 8°C was the ambient temperature. Fish in the 18°C treatment were acclimated by placing the demonstrator chamber into a water bath and increasing its temperature from 8°C to 18°C over a 72 h period. This was sufficiently long enough to avoid any shock, and no adverse effects were seen in the fish either during or after the study. Fish in the 8°C treatment were also placed in the water bath (with the heater switched off) for 72 h, and were not subjected to any temperature increase. The demonstrators were held in demonstrator chambers measuring 30 × 15 × 30 cm, 20 cm water depth (electronic supplementary material, figure S4). The layout of the experimental arena and demonstrator chambers was otherwise identical to that described in figure 1. During the trial, we presented a focal fish with a binary choice of one group of demonstrators in 8°C water and another group in 18°C water. The demonstrator tanks were partially insulated, and water temperature was observed to fall by approximately 3°C over the 25 min duration of the trials.

*Strike rate*. Here, we presented demonstrators with prey-like stimuli in order to induce feeding strikes. As prey odour cues alone may cause fish to become more active (as increasing activity can increase encounter rates with prey), we used a non-food visual stimulus designed to elicit a feeding response upon presentation, but without any residual odour cues which may persist beyond the presentation of the stimulus. Both *G. aculeatus* and *P. pungitius* readily attack red objects, the result of a receiver bias probably evolved for foraging for carotenoid-rich prey [40]. We exploited this by using a red laser pointer to project a dot of red light into the base of the feeder unit. The base of the feeder was angled at 45^o^, facing the front of the feeder and was coloured white to maximize the visibility of the red dot. One laser pointer was mounted 30 cm above each feeder. They were operated manually. The pen was switched on for 10 s to simulate a ‘prey delivery’, during which time the fish could ‘attack’ the red dot. The dot in one feeder was not visible to the focal fish in the experimental arena, nor to the stimulus fish held at the other feeder. During the trial, we presented a focal fish with a binary choice of moving towards one group of demonstrators which were exposed to the red laser dot ‘prey’ stimulus once and another group which were exposed to it three times. A pilot study confirmed that the strike rate was higher when the laser pointer was presented three times compared with when it was presented once, while activity rate did not vary (electronic supplementary material, part S3, figure S3). A colourless perforated barrier was used to divide the chamber into two unequally sized sections of 10 × 15 cm and 20 × 15 cm (electronic supplementary material, figure S4). The feeder and the fish were held in the smaller section. This allowed us to hold shoal density and distance from feeder relatively constant between the two stimulus groups.

The procedure for the PI treatment, in which the observers were tested after the demonstrators had been removed, was identical to the standard PI assay but without prey delivery. The procedure for the real-time choice treatment, in which the observer was tested in the presence of the demonstrators differed slightly. Here, no simulated prey (i.e. red laser dot stimulus) was applied during the demonstration phase. The observer was exposed to demonstrators in the absence of any stimulus during this period. Instead, the simulated prey stimulus began in the choice phase, 30 s after the observer was released from its holding unit, with simulated prey exposures being applied to the rich patch demonstrator group after 30 s and again on 150 s, and to both groups after 470 s.

*Shoal cohesion.* Here, we presented cohesive and dispersed groups of demonstrators to observer fish to determine whether group cohesion was a cue used by observers when selecting patches. We used colourless perforated plastic barriers to divide the demonstrator chamber (measuring 30 cm wide × 15 cm long × 30 cm tall, 20 cm water depth), into five compartments, each measuring 6 × 15 cm (electronic supplementary material, figure S4). One demonstrator chamber held a dispersed shoal, with one fish in each outermost compartment and one in the centre, with the compartments in between empty. This held fish between three and five body lengths apart. The other held a cohesive shoal. All three fish were placed in the central section, and were never able to be more than two body lengths apart. No feeders were present in the demonstrator chamber.

*Distance from feeder.* Here, we tested whether demonstrator proximity to feeder affected observer patch choice. We used colourless perforated plastic barriers to divide the demonstrator chamber (measuring 30 cm wide × 15 cm long × 30 cm tall, 20 cm water depth), into three compartments, each measuring 10 × 15 cm (electronic supplementary material, figure S4). A feeder was present in the outer bottom corner of each chamber. One chamber held fish in the same compartment as the feeder, within four body lengths of it at all times. The other held fish in the furthest compartment, at around at least seven body lengths away from the feeder.

*Statistical analysis*. As in Part 1, we used count data on time allocation (time in ‘rich’ patch − time in ‘poor’ patch goal zones) derived from point sampling the location of the fish at 6 s intervals. Here, ‘rich’ refers to the high activity/high strike rate/cohesive/close to feeder treatment and ‘poor’ low activity/low strike rate/dispersed/distant from feeder treatment. Data were analysed using GLMs with a Poisson distribution, with species and treatment (real time or PI-use) as fixed factors with the interaction between these also specified. We performed a separate analysis for each of the four demonstrator cue treatments.

#### 5.1.2. Results

**Figure 5.**
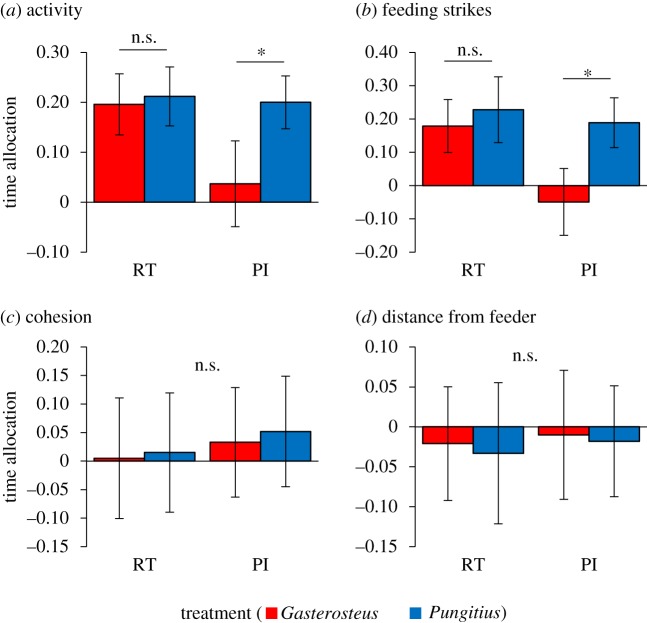
Mechanism: observers of both species were attracted to demonstrator shoals that were more active (*a*) and that performed more feeding strikes (*b*) compared with less active shoals and shoals with lower feeding strike rates. Such preferences were exhibited by both *Pungitius* and *Gasterosteus* when patches were chosen with demonstrators present (real time, RT), but only by *Pungitius* when patch choice occurred after demonstrator removal (public information, PI). Neither species showed any attraction towards more or less cohesive demonstrator shoals (*c*) or for shoals that were closer to or further from the feeder (*d*). Time allocation refers to the mean proportion of time spent in rich patch − time in poor patch, ±95% CI. *p < 0.05.

*Activity*. We saw differences between species, an effect of treatment and an interaction between the two (*F*_1,74_ = 6.67, *p* = 0.011; *F*_1,74_ = 6.08, *p* = 0.016; *F*_1,74_ = 4.49, *p* = 0.037, figure 5*a*). Both species showed a clear preference for the goal zone closest to the more active demonstrators in the real-time treatment (i.e. while these were still visible), but *P. pungitius* spent more time in the active group goal zone than did *G. aculeatus* in the PI treatment, with the latter showing no preference for either goal zone.

*Feeding strike rate*. Here, we saw differences between species, and an effect of treatment and an interaction between the two (*F*_1,74_ = 11.51, *p* = 0.001; *F*_1,74_ = 9.96, *p* = 0.002; *F*_1,74_ = 4.99, *p* = 0.028, figure 5*b*). Both species showed a clear preference for the goal zone closest to demonstrators performing the higher feeding strike rate in the real-time treatment, with only *P. pungitius* spending more time closer to this group in the PI treatment.

*Group cohesion*. Here, we saw no preference for the more or less cohesive demonstrator group by either species under either treatment (species: *F*_1,74_ = 0.07, *p* = 0.787; treatment: *F*_1,74_ = 0.37, *p* = 0.545; interaction: *F*_1,74_ = 0.01, *p* = 0.933, figure 5*c*).

*Distance from feeder*. Again, we saw no preference for the more or less cohesive demonstrator group by either species under either treatment (species: *F*_1,74_ = 0.06, *p* = 0.807; treatment: *F*_1,74_ = 0.10, *p* = 0.750; interaction: *F*_1,74_ = 0.01, *p* = 0.961, figure 5*d*).

### 5.2. The relative importance of activity and feeding strike rate

The experiments described above revealed that the activity levels and feeding strike rate of demonstrators increased with increasing prey provision, and that observers of both species were attracted to more active demonstrators and to demonstrators performing more feeding strikes when they were allowed to approach them in real time, while *P. pungitius* (but not *G. aculeatus*) also remembered the locations of these demonstrator groups after they had been removed. In this experiment, we compared the relative importance of these cues, presenting observers with a choice of approaching either of two sets of demonstrators that were either highly active, but which were performing a low feeding strike rate (high temperature/one prey stimulus exposure), or had lower activity, but a higher feeding strike rate (low temperature/three prey stimulus exposures). Activity and strike rate were manipulated using the water temperature and the red laser pointer prey stimulus procedures described previously.

#### 5.2.1. Methods

Observers were presented with two sets of demonstrators, one that was less active but performed a higher feeding strike rate and one that was more active but which performed fewer strikes. This was achieved using the water temperature and prey stimulus approaches described above. Before conducting the experiment proper, we first performed a pilot experiment in order to quantify demonstrator activity and strike rate under these conditions. This work, described in the electronic supplementary material, part S4 and figure S5, demonstrated that this approach was successful in manipulating demonstrator behaviour as desired.

With the experimental design validated, we went on to conduct observer choice tests. These treatments were performed for both species, both in real time and after the demonstrators had been removed, simulating PI. We performed 25 replicates for each species and treatment group combination, running trials in October–December 2009. The procedures and set-up were otherwise the same as that described above for the activity and feeding strike experiments in (§5.1).

*Statistical analysis*. We used count data on time allocation (time in high activity/low strike patch − time in low activity/high strike patch goal zones) derived from point sampling the location of the fish at 6 s intervals. Data were analysed using a GLM with a Poisson distribution, with species and treatment (real time or PI-use) as fixed factors with the interaction between these also specified.

#### 5.2.2. Results

**Figure 6.**
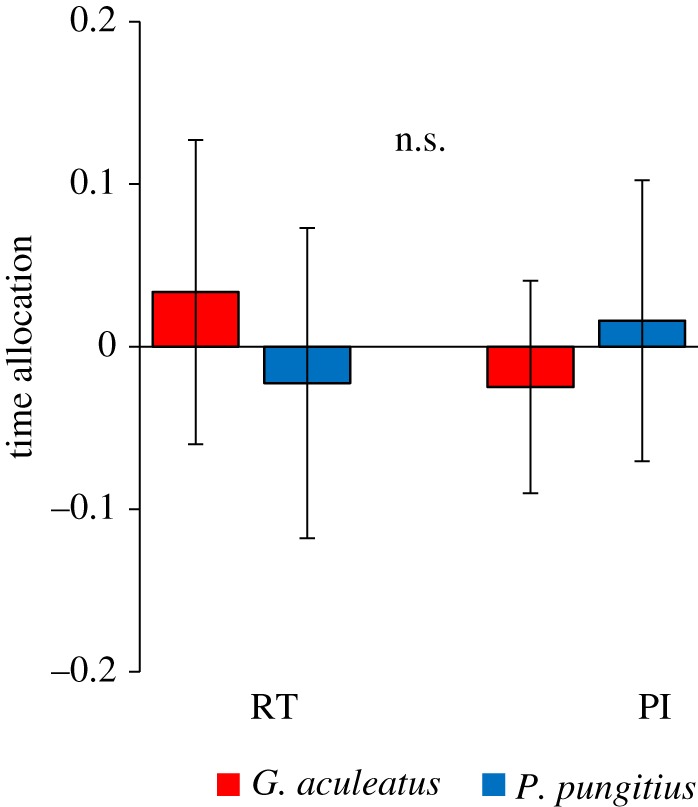
Relative importance of demonstrator activity and strike rate in shaping observer prey patch preferences. Time allocation refers to time in high activity, low strike rate goal zone − time in low activity, high strike rate goal zone (mean proportion ± 95% CI). RT refers to the real-time treatment, where the demonstrators were visible to the observer as it moved between patches, and PI refers to the PI-use treatment, where the observer moved between patches after the demonstrators had been removed from sight.

Neither species was seen to show any preference for either demonstrator group, either in real time or under PI-use conditions (GLM: species *F*_1,96_ = 0.11, *p* = 0.74; condition *F*_1,96_ = 0.17, *p* = 0.68; interaction *F*_1,96_ = 0.71, *p* = 0.40, figure 6).

### 5.3. Animated demonstrators

Through careful manipulations in §§5.1 and 5.2, we were able to identify activity and feeding strike rate cues as important components of PI. In practice, it is difficult using live demonstrators to investigate one behaviour or cue while holding others constant. To overcome this, we developed, piloted and used computer-generated three-dimensional animated demonstrator *P. pungitius* in PI-use experiments, allowing us to manipulate activity and/or strike rate in simulated demonstrator shoals without inadvertently generating variation in other cues, enabling us to further explore the social transmission of PI. These experiments were performed for *P. pungitius* only. The aims of these experiments were to develop a system for studying PI-use that allowed for greater precision and consistency than that achievable using live demonstrators [41].

#### 5.3.1. Methods

**Figure 7.**
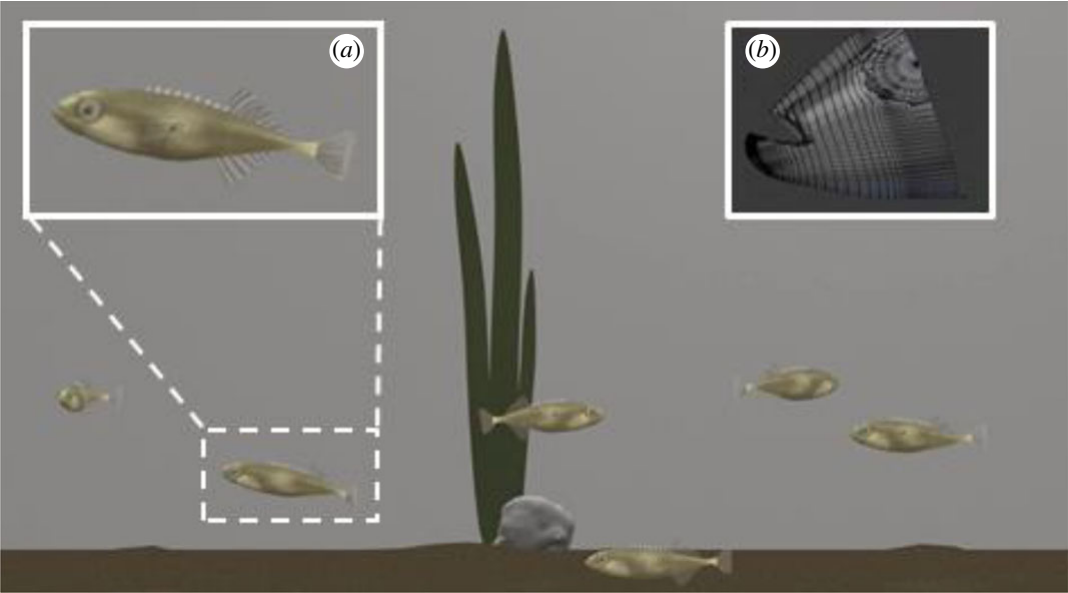
Computer-generated animated sticklebacks used in experimental investigations of the PI-use mechanism. Inset panels show (*a*) close up of animated *P. pungitius* and (*b*) screengrab of construction of animation.

The animations were built using Blender (www.blender.org), a free open-source three-dimensional content creation suite (electronic supplementary material, part S5; figure 7). The animated stimuli were validated in a pilot experiment which confirmed that real observers responded and were attracted to them (electronic supplementary material, part S5 and figure S6). The animated stimuli were then presented to the test subjects using a modified version of the PI-use binary choice test. We performed seven treatments in which observers were given a binary choice between patches demonstrated by animated demonstrators that varied in their activity rate, feeding strike rate, distance from feeder, shoal cohesion (one each in the *x* and *y*-axes), or position in the water column and a final treatment in which all of these cues were presented together.

The experimental arena was designed to be similar to the standard PI test arena used throughout. The observer was housed in a 30 × 30 × 30 cm glass aquarium. On either side of this, we placed two 30 cm (screen width) CRT monitors, each attached to a laptop. The monitors were positioned so that their screens faced into the observer tank, and were placed 1 cm away from its glass walls. The monitors were used to display the stimulus videos to the test subject, held within the observer tank [42]. A 10 × 10 cm base, 25 cm tall perforated, colourless plastic holding unit was placed in the centre of the observer tank. The trials were filmed from the side, through the wall of the observer tank, using a high-definition camera mounted on a tripod.

Each trial consisted of three phases: habituation, demonstration and testing. In the habituation phase, a single observer fish (the test subject) was placed in the holding unit and presented with empty images of the animated scene on both monitors (i.e. one that contained the background images, but no animated fish) for 10 min. In the demonstration phase, the animated demonstrators appeared onscreen and the two stimulus videos (described below) were played simultaneously. The videos lasted 10 min. At the end of the videos, the animated fish swam off the side of screen, leaving only the empty tank scene. After 1 min, the holding unit was carefully raised and removed, releasing the observer and beginning the test phase. This lasted for a further 5 min, during which we recorded the location of the observer fish at 6 s intervals, noting whether it was within 8 cm (the goal zone) of either of the two monitors.

We modified the behaviour of the animated fish in order to produce seven treatments that captured the demonstrator behaviours seen to vary with prey delivery rate in the trials conducted with real fish, plus an additional behaviour that we did not previously consider, demonstrator vertical position in the water column. Sample sizes vary between treatments because trials in which the fish froze during the test phase were excluded.

*Activity rate (n = 24)*. Activity rate was modified such that the animated demonstrators in the higher activity simulation swam at a rate of 15 body lengths per minute, while those in the lower activity simulation swam at 10 body lengths per minute. This was comparable with the variation in the mean swimming speeds seen between the live demonstrators in the rich and poor feeder conditions (electronic supplementary material, figure S1).

*Feeding strikes (n = 14)*. Feeding strikes were simulated by incorporating short bursts of movement directed towards the feeder unit into the movement paths of the fish. We inserted a striking cycle of two successive strikes per fish at 90 and 540 s in the 600 s long animation representing the poor patch, and at 90, 180, 270, 360, 450 and 540 s in the rich patch animation, simulating a rich : poor food yield ratio of 3 : 1.

*Distance from feeder (n = 17)*. We manipulated proximity to feeder in the animated demonstrator stimulus videos by restricting the fish to swim in the half of the scene that contained the feeder (rich patch simulation) or in the opposite half (poor patch simulation).

*Shoal cohesion.* Two treatments were conducted, one in which the fish were animated as cohesive or dispersed in the *x*-axis (*n* = 21), and one in which they were animated as cohesive or dispersed in the *y*-axis (*n* = 18). The first set of animation, cohesion on the *x*-axis, had the fish freely swimming through the animated tank in the poor patch simulation, and constrained to a column in the central third of the tank in the rich patch. The animations testing the cohesion between individuals on the *y*-axis had the fish swimming through the whole tank in the poor condition, while constrained to a row occupying the central third of the tank in the rich condition.

*Water column position (n = 19)*. *Pungitius pungitius* sticklebacks typically feed on or near the substrate. We therefore reasoned that when in a rich patch, most of their time would be spent in the lower part of the water column. To investigate this, fish were restrained to the upper third of the water column in the poor patch simulation and to the lower third in the rich patch simulation.

*All cues (n = 18)*. Finally, we ran a treatment in which all of these cues were presented together. In the poor patch simulation, fish were randomly distributed within the water column and in the lateral plane, feeding at two deliveries per animation, and swam at low speed through the tank. The rich patch animation showed a shoal moving exclusively within the lower third and the feeder half of the tank, swimming rapidly and striking six times during the simulation, as described above.

*Statistical analyses*. In each trial, we determined the number of point samples (6 s intervals, 50 points in total) spent in each of the two goal zones next to each animated demonstrator shoal. These were compared for each of the seven cue pairings using paired samples *t*-tests.

#### 5.3.2. Results

**Figure 8.**
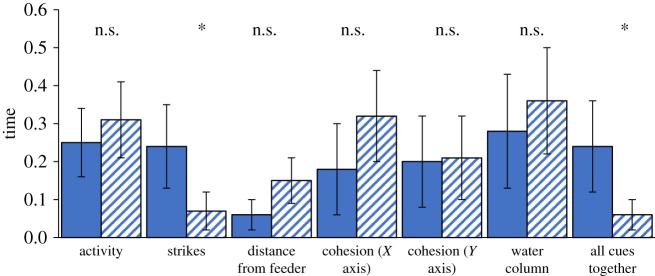
Response of fish to animated stimuli displaying different combinations of PI cues. The proportion of time spent in rich (solid bars) and poor (hatched bars) goal zone (mean ± 95% CI). Activity: faster versus slower swimming speeds; strikes: more versus fewer feeding strikes; distance from feeder: closer or further; cohesion: fish were closer together or further apart in the *X* and *Y*-axes, respectively; water column: fish were lower or higher from the bottom of the scene. In each case, the former stimulus corresponds to ‘rich’ patch and the latter to ‘poor’ patch feeding behaviour. See main text for further details. n.s., no significant difference. *p < 0.05.

In the feeding strikes comparison, observers spent more time in the goal zone of the group that performed more feeding strikes (paired samples *t*-test: *t* = 2.12, d.f. = 14, *p* = 0.05). Subjects also spent more time in the goal zone of the rich patch demonstrator group when all cues were presented together (*t* = 2.44, d.f. = 18, *p* = 0.02). However, subjects showed no preference for either goal zone for any other cue comparison (activity: *t* = −0.64, d.f. = 24, *p* = 0.53; distance from feeder: *t* = −1.95, d.f. = 17, *p* = 0.07; cohesion (*x*): *t* = −1.17, d.f. = 21, *p* = 0.25; cohesion (*y*): *t* = −0.08, d.f. = 18, *p* = 0.93; water column: *t* = −0.59, d.f. = 19, *p* = 0.56; figure 8).

The observer preference for approaching the high strike rate demonstrator group mirrors our finding with the live demonstrators (§5.2); however, our failure to observe a preference for the more active demonstrator group in this experiment is at odds with the finding in the corresponding live demonstrator experiment. It may be that fish feeding at higher rates not only swim more rapidly; their activity may differ in more subtle ways too, such as in frequency of turning rate, acceleration bursts or in posture, variables that we did not account for in our animations. It would be informative to extend this work by extracting data on such cues from observations of real fish and incorporating them into more realistic animated stimuli.

## 6. Function: does PI-use reflect general learning ability?

### 6.1. Methods

Does PI-use function as a form or component of social foraging, or does it reflect and covary with a general difference in how stickleback species learn? In this final series of experiments, we investigated the specificity of the species difference in learning seen in §§3 and 5, by exploring whether there were other differences in the social or asocial learning of *P. pungitius* and *G. aculeatus*. This would allow us to determine whether the basic PI-use findings reflected an ‘ecological specialization’ for a particular form of social learning in *P. pungitius*, or a more general species difference in learning capabilities. In four experiments, we compared the performance of both species in the standard PI test, a local enhancement test (a similar test in which the fish were allowed to select a goal zone in real time, while the demonstrators were still present and feeding), and two asocial learning tasks testing operant conditioning (learning to swim left or right in a maze to receive a food reward) and classical conditioning (learning to associate a colour cue with food).

We collected fish of both species (35–40 mm long) from Melton brook, UK (electronic supplementary material, table S2). Within each trial, the test fish and demonstrators were size matched to within 2 mm of each other. We tested 20 fish per species in each of the four tasks, in batches of 10 fish, consisting of five *G. aculeatus* and five *P. pungitius* each. In between trials, each fish was held in its own 45 l aquarium. Two companion conspecifics measuring 25 mm long were added to each tank. This helped to minimize stress in the test fish and the size difference ensured that it could be easily recognized. Experiments took place between April 2007 and October 2008.

*PI-use*. Fish were tested in the standard PI-use assay described above. The procedure was the same except that here each fish was tested multiple times, in order to obtain a measure of behaviour that was comparable to those obtained from the asocial learning tasks, described below. Each fish was tested 10 times on alternate days, over a 20-day period. We only recorded first choice of goal zone as a response variable, and trials were ended as soon as the fish had entered one of the goal zones. Once the fish had a selected either goal zone, three bloodworms were provided in the goal zone of the rich patch, and the fish was left in the tank for 15 min to eat these. The location of the rich goal zone was randomized between trials.

*Local enhancement*. We tested whether subjects were attracted to one of two groups of conspecifics that were feeding at the higher rate in real time. This test would enable us to determine whether differences in PI-use by the two species encompassed differences in learning, or were due to differences in the perception of feeding-related cues. Fish were tested in a modified version of the PI assay. As previously, the rich patch demonstrators were fed three times, while the poor patch group were fed once, receiving two blanks of tank water on the occasions that they were not fed, but while in the PI assay, the demonstrators were removed from view during the choice phase of the assay, here they were retained during the choice phase. The observer was held in the holding unit for the first 4 min of the demonstration phase, during which time the rich patch demonstrator group were fed twice. The observer was released on the 4 min mark, with both the rich and poor patch demonstrators receiving a food delivery 1 min later. Trials were ended as soon as the observer entered one of the goal zones, and food was provided in the rich patch goal zone (even if the observer had entered the poor patch goal zone, as described above). As with the PI-use assay, each fish was tested 10 times on alternate days, over a 20-day period and we only recorded first choice of goal zone as a response variable. The location of the rich goal zone was randomized between trials.

*Asocial learning: operant conditioning*. Fish were tested in a T-maze. Each arm was 50 cm long by 20 cm wide and 20 cm deep. The final 8 cm of each of the upper arms was designated as a goal zone. The maze was constructed from black plastic and filled with sand to a depth of 1 cm. The water depth was 15 cm. Fish were randomly allocated to be trained to swim into the left or right arms to find food. Each fish received 7 days of pre-training, once per day. Fish were trained and tested individually. At the beginning of each session, 10 dead bloodworms were added to the far end of either the left or right arm of the maze, with the rewarded arm randomly predetermined for each subject but consistent across trials. The subject was then placed in the lower arm of the maze, within a 10 × 10 cm base, 25 cm tall perforated colourless plastic tower. It was held within the tower for 15 min in order to acclimate. Following this, it was released from the tower and allowed to move freely within the T-maze for 30 min, before being returned to its holding tank. Within each period, all subjects found the prey, and ate some of it (no fish ate all of the 10 prey that were provided).

This training period was followed by the test period. Each fish was tested 10 times on alternate days, over a 20-day period. The acclimation and testing procedure was performed as above, with the modification that food was initially absent from the T-maze. As soon as the fish entered one of the goal zones, we added three bloodworms to the goal zone of the correct arm (that is, the arm to which the fish was being trained, even if it entered the wrong arm during the trial). Food was not added until after the decision had been made to ensure that its choice was not affected by odour cues from the prey. The fish was then left for 15 min to feed. This ensured that the fish continued to receive positive reinforcement over the duration of the testing phase. We collected data on three response variables: the number of trials before the fish entered the target goal zone first three times in a row, four times in a row and the number of correct goal zone entries in the final five test trials.

*Asocial learning: classical conditioning*. Fish were trained using the same T-maze apparatus and procedure as above, only here they were trained to swim into the arm with a particular coloured wall. Removable blue or yellow panels were placed along the length of the back wall of each upper arm. Fish were randomly assigned to be trained to blue or yellow. The coloured panels were in turn randomly assigned to either arm for each training trial or test trial. The training and test trials were otherwise conducted exactly as above. We collected data on three response variables: the number of trials before the fish entered the target goal zone first three times in a row, and four times in a row, and the number of correct goal zone entries in the final five test trials.

*Statistical analyses*. For each of these tasks, test subjects were tested 10 times and we compared the performances of the two species. We compared the number of trials in which each fish entered the rich patch first in the PI-use and local enhancement tasks using Mann–Whitney *U*-tests. For the operant and classical conditioning tasks, we compared the number of trials taken for the fish to make three correct choices, i.e. select the rewarded arm of a T-maze, and, as a more stringent measure, four correct choices in a row, again using Mann–Whitney *U*-tests. In order to determine whether test subjects tended to visit the rewarded patch first more than would be expected by chance had no learning occurred, we used *χ*^2^ tests to compare the number of trials in which the fish visited the rewarded patch against a null expected visiting rate of five out of the 10 trials.

### 6.2. Results

**Figure 9.**
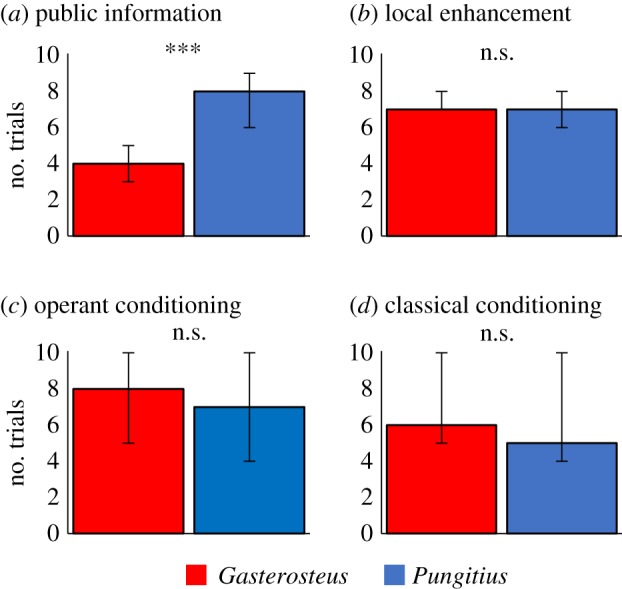
Function: while *Pungitius* and *Gasterosteus* differed in PI-use, no other species differences in learning were found. The number of trials out of 10 (median ± quartiles) in which fish selected the rich patch when choosing (*a*) after the demonstrators had been removed (public information), and (*b*) while the demonstrators were still present (real-time patch choice, or local enhancement). The number of trials until the rewarded arm was chosen three times in a row (median ± quartiles) for (*c*) learning to swim left or right in a T-maze to receive food (operant conditioning), and (*d*) learning to associate a colour with a food reward (classical conditioning). ***p < 0.005.

Consistent with earlier experiments, we saw that *P. pungitius* selected the rich patch more frequently than did *G. aculeatus* in the PI-use task (Mann–Whitney *U*: *U*_20,20_ = 27, *Z* = −4.72, *p* < 0.001), but we found no other differences in the learning abilities of the two species (figure 9). Both species entered the rich patch more times than would be expected by chance in the local enhancement treatment (*U*_20,20_ = 187, *Z* = −0.72, *p* = 0.71). In the asocial operant conditioning task, we saw no differences between the two species in the number of trials until three or four correct goal zones in a row were selected, or the number of correct goal zone choices in the final five trials (Mann–Whitney *U*-tests: *U*_20,20_ = 170, *Z* = −0.84, *p* = 0.39, *U*_20,20_ = 156, *Z* = −1.25, *p* = 0.21, *U*_20,20_ = 146, *Z* = −1.51, *p* = 0.14, respectively). Over the 10 trials, both species tended to visit the rewarded patch first more than would be expected by chance had no learning occurred (*χ*^2^ test: *G. aculeatus χ*^2^ = 3.20, d.f. = 1, *p* = 0.07; *P. pungitius χ*^2^ = 7.20, d.f. = 1, *p* = 0.007). In the classical conditioning task, the number of trials until three or four correct goal zones in a row were selected and correct goal zone choices in the final five trials did not differ (*U*_20,20_ = 172, *Z* = −0.76, *p* = 0.45, *U*_20,20_ = 151, *Z* = −1.41, *p* = 0.16, *U*_20,20_ = 148, *Z* = −1.45, *p* = 0.15). Again, over the 10 trials, both species tended to visit the rewarded patch first more than would be expected by chance, providing clear evidence of learning (*χ*^2^ = 5.00, d.f. = 1, *p* = 0.025 in both cases).

Combined with the evidence that *G. aculeatus* are capable of other forms of social learning, expressed in cooperative anti-predator behaviour [43], kin recognition and familiarity [44,45], and social foraging [39,44–48] contexts, our findings imply that the observed difference between *P. pungitius* and *G. aculeatus* in learning is specific to public information concerning food patch quality.

## 7. Discussion

Over series of experiments, we have investigated the phylogenetic distribution, development, mechanisms and function of PI-use across a family of fishes. PI-use varied between species. It was seen in most populations of *Pungitius* and in some of *C. inconstans*, but not in any of the multiple populations of *G. aculeatus* nor either of the single populations of *A. quadracus* or *S. spinachia* that we tested. Within *P. pungitius*, PI-use was not seen to be readily influenced by ontogenetic experience: neither rearing density or prey distribution were seen to affect it. The activity levels and feeding strike rate of demonstrators seem to be key components of PI, attracting observing *P. pungitius* and, when allowed to move directly towards the demonstrators as they presented these cues, *G. aculeatus* too. Finally, the species difference in PI-use between *P. pungitius* and *G. aculeatus* did not appear either to stem from differences in demonstrator behaviour (because both species were attracted to demonstrators in real time) nor did it reflect some general difference in learning, because the two species did not differ when tested in other learning tasks.

Why is PI-use seen in some species but not others? Coolen *et al*. [24] reported a similar species difference in PI-use between *P. pungitius* and *G. aculeatus*, albeit from a single population of each, collected from the same location. They suggest that vulnerability to predators might be an important factor. *Gasterosteus aculeatus* have larger spines than do *P. pungitius*, and these provide protection from gape-limited predators, potentially leaving them less vulnerable to being captured [49]. Coolen *et al*. [24] argued that PI-use might have evolved in *P. pungitius* because it allows them to gather information about the distribution of resources without the need to search and sample their surroundings directly, a functional explanation, which might reduce the risk of encountering a predator. They reported that *P. pungitius* were more likely to observe demonstrators from cover than were *G. aculeatus*, and other studies have found that *P. pungitius* spend more time in cover than do *G. aculeatus* too [50,51]. Our findings are consistent with this account, though they do not explicitly support it and other explanations are possible. That PI-use was seen in *Pungitius* and (some) *Culaea*, two lineages derived from a common ancestor [52] might suggest that this behaviour evolved before they diverged, though again this is not something for which we have direct evidence. A second experiment compared PI-use in a subset of *P. pungitius* and *G. aculeatus* populations from the UK, for which we had basic information on predation pressure for separate channels occupied by each population. Here, we found no evidence for an effect of predation on PI-use: PI-use was not greater in populations of either species when inferred predation pressure was higher, as it might be predicted to be if PI-use is both labile and reduces risk of being captured [24].

In the second part of this project, we investigated the effects of rearing environment upon the development of PI-use in *P. pungitius*. Neither rearing density nor the distribution of food had any effect upon PI-use in fish raised under these conditions for five months from juveniles to subadults. This finding suggests that plasticity in PI-use is not readily inducible. At the start of these experiments, the fish were already free-swimming and free-feeding juveniles and it is possible that they had already been exposed to some critical window for the development of PI-use, for example, an opportunity to learn an association between the feeding behaviour of others and the presence of food. Nevertheless, the fact that five months exposure to consistently different social environments and social foraging conditions did not induce variation in PI-use was surprising. In a separate experiment, Webster & Laland [39] investigated how experience shaped social information use in a novel foraging task in adult *P. pungitius* and *G. aculeatus*, finding that in both species, experience of both completing the foraging task themselves and of seeing others do so too was necessary for the fish to respond to social information about the task later on. This finding suggests that, in some contexts, social information use in these fish is influenced by their social experiences, and clearly contrasts with the findings of the PI-use ontogeny experiments presented here. Other experiments report comparable findings in other taxa, including birds [53,54] and social insects [55]. Recent debate concerns the extent to which social learning, which includes PI-use, is mechanistically distinct from other forms of learning. In particular, some have argued that many instances of social learning, including PI-use by fishes, can be explained using purely associative learning [4]. These assertions have been argued by others to be premature, given the relative lack of research directly addressing this question [56], and the data presented in this study in both the ontogeny experiment, the species differences seen in the phylogeny experiments and in the function experiments discussed below, would seem to suggest that PI-use in sticklebacks cannot adequately be explained in purely associative terms.

A comprehensive set of experiments into the cues involved in PI-transmission identified two demonstrator behaviours, activity rate and feeding strike rate as the most important in attracting observers to the richer prey patch. These behaviours are necessarily correlated with prey yield, because competing foragers speed up and perform more strikes as they find more food items. These cues are almost certainly produced inadvertently as a by-product of foraging, though it would be interesting to determine whether foraging sticklebacks are sensitive to their audience, adjusting the foraging behaviour accordingly as has been reported in other species (e.g. [57]). These findings account for the ‘mechanism’ of how PI is transmitted in a physical sense, by identifying the behavioural cues that covary with prey delivery and to which the observers attend. Insight into the cognitive mechanisms underlying PI-use comes from earlier studies. Webster & Laland [28] showed that the rich patch location is learned by *P. pungitius*, ruling out a plausible alternative explanation, that the travel direction is simply affected by the observer orienting towards the demonstrators without the observer actually learning anything. They showed that the direction the observer was facing at the moment of release did not predict which of the two patches that it visited first, and that fish that were forced to swim through a chicane, which oriented them away from the rich patch, were still able to find their way there. Another study by these authors [29] investigated what is learned by the observer. Observers learned the location of the rich patch, but did not learn to associate demonstrator feeding behaviour with conspicuous landmarks placed near the patch. When the landmarks associated with a rich and poor patch were switched, the fish moved to the location where they saw the demonstrators feeding at a greater rate, and not the location of the now-relocated landmarks that were paired with the patches. When access to the original locations of the rich and poor patches was prevented and new landmarks in new locations were presented, the fish showed no preference for either. With reference to Coolen *et al*. [24], but applicable here too, Heyes & Pearce [4] provide an associative account of how PI might operate. They suggest that fish that have foraged socially might learn an association between feeding themselves and seeing others feed. This is very plausible, but alone cannot account for the species differences in PI-use reported by Coolen *et al*. [24] or here. This difference is unlikely to be due to differences in what the two species can see, because both were attracted to the group feeding at the higher rate in real time. Differences in demonstrator behaviour can be ruled out too: Coolen *et al*. [24] showed that *P. pungitius* could still locate the rich patch when *G. aculeatus* demonstrators were used, but not vice versa. Future work might focus upon the neurophysiology of PI-use, potentially providing insight into the molecular mechanisms underlying the differences seen between these two species with respect to this behaviour.

Our final set of experiments investigated different forms of learning in *P. pungitius* and *G. aculeatus*. While the same differences in PI-use seen in other parts of the study persisted, we saw no differences between the two species in other forms of learning, specifically classical and operant conditioning. Other studies have found correlations between social and asocial learning, for example, in pigeons (*Columba livia*) [58] and in zebra finches (*Taeniopygia guttata*), where song-learning and asocial learning when foraging were correlated [59]. Heyes [60] suggests that covariation between asocial and social learning supports the hypothesis that they both are controlled by the same mechanism. In our experiment, we did not look to see if different forms of learning were correlated, that is, whether better PI-users also performed better at operant or classical conditioning tasks, for example, so we cannot address this question directly. Given the species-level difference in PI-use, but not in other forms of learning though, it seems unlikely that PI-use simply reflects general learning ability. The evidence presented in this set of experiments suggests that PI-use is mechanistically distinct from other forms of learning. We suggest that PI-use represents an ‘ecological specialization’, functioning as a means of effectively gathering information during social foraging [24,61–63].

By addressing all four of Tinbergen's [11] questions, our study contributes to understanding of how selection shapes the acquisition and use of information by animals. First, the social learning capabilities of *Pungitius* appear to be ecologically specialized, being restricted to a particular mechanism (PI-use) deployed in a foraging context. This fits with other findings (e.g. the honeybee (*Apis melifera*) waggle dances transmit information about food and nest sites, but not other information [64]), implying that the cognitive abilities of animals can be tailored by selection to be functional in the domains in which those abilities evolved, but not necessarily other domains. Second, the absence of evidence for general differences in learning in *Pungitius* compared with *Gasterosteus*, alongside analyses of mechanism, suggests that selection may have fine-tuned the perceptual, motivational and/or information-processing capabilities of *Pungitius*, rather than incrementing learning. This is consistent with Shettleworth's [65] conclusion that *what* an animal learns may be ecologically specialized and therefore vary among species, but *how* animals learn (at least, at the level of the underlying associative processes) appears to be broadly similar across diverse taxa. Third, PI-use has been seen in distantly related animal groups (humans, some birds, sticklebacks) apparently in response to particular ecological conditions [15,66]. This may be indicative of a general pattern, suggesting that animal cognition can evolve independently and mosaically in diverse taxa through convergent selection [67]. Given their prominence as a model system in behavioural and evolutionary research, and their amenability to experimental analysis [68,69], the study of PI-use in sticklebacks generates exciting opportunities to investigate the mechanisms underlying this behaviour further, including at genetic, neural and physiological levels.

## Supplementary Material

Webster et al. PI-use_ESM

## Data accessibility

The data used in this study are available via the Dryad Digital Repository: https://dx.doi.org/10.5061/dryad.0cq3rm5 [70].
