# Supplementary material for: A four-questions perspective on public information use in sticklebacks (Gasterosteidae)
Source: R Soc Open Sci. 2019 Feb 20;6(2):181735. doi: 10.1098/rsos.181735 (PMC6408396; doi:10.1098/rsos.181735)
Supplement: Webster et al. PI-use_ESM [file rsos181735supp1.docx]

ESM for

**A Four-Questions Perspective on Public Information Use in Sticklebacks (Gasterosteidae)**

Mike Webster, Laura Chouinard-Thuly, Gabor Herczeg, Jun Kitano, Riva Riley, Sean Rogers, Michael D. Shapiro, Takahito Shikano, Kevin Laland

Correspondence to: mike.m.webster@gmail.com

Contents:

|  |  | Page |
| --- | --- | --- |
| Text S1 | Pilot experiments: quantifying demonstrator behavior at different feeding rates | 2 |
| Figure S1 | Demonstrator behavior under different prey delivery rates | 5 |
| Text S2 | Demonstrator activity: pilot experiment | 6 |
| Figure S2 | Demonstrator activity: pilot experiment results | 7 |
| Text F3 | Demonstrator strike rate: pilot experiment | 8 |
| Figure F3 | Demonstrator strike rate: pilot experiment results | 9 |
| Figure S4 | Demonstrator behavior cues diagram | 10 |
| Text S4 | Relative importance of demonstrator strike rate and activity cues- pilot | 11 |
| Figure S5 | Relative importance of demonstrator strike rate and activity cues- pilot results | 12 |
| Text S5 | Animated demonstrators: building and piloting | 13 |
| Figure S6 | Animated demonstrators: pilot results | 16 |
| Table S1 | Stickleback populations tested in the Gasterosteidae species comparison | 17 |
| Table S2 | Stickleback populations tested in the high- and low-predation comparison | 19 |

**S1. Pilot experiments: quantifying demonstrator behavior at different feeding rates**

We quantified the behavior of groups of demonstrators feeding at different rates, in order to determine which aspects of their behavior might co-vary with their feeding rate. Such behavioral variation might facilitate prey patch quality estimates in observers, an idea we go on to test explicitly, below. We established groups of three fish within a test tank measuring 30cm x 15cm x 30cm (height), 20cm water depth, identical to the demonstrator chambers of the test tank used in the standard PI binary choice assay, described in Figure 1. A feeder unit was placed in the bottom left corner. We ran three feeding treatment groups, in which the fish received either 0, 1 or 3 prey items in a 6-minute period. The 1- and 3-prey groups represented the high and low quality feeder treatments in the standard PI binary choice assay. The 0-prey group allowed us to collect baseline data for comparison. In the 3-prey group, prey were added after 0, 2 and 4 minutes. In the 1-prey group prey was added after 2 minutes only. At the intervals in which no prey were added in the 0 and 1 prey treatments a blank of 1cm^3^ water containing no prey was added. Fish were allowed to settle for 10 minutes before the 6-minute feeding section began. There were 12 groups per treatment, per species.

We recorded the following traits: activity, feeding strike rate, shoal cohesion and distance from the feeder. Activity rate, expressed in body lengths travelled per minute, was quantified using Logger Pro tracking software (Vernier Software & Technology, Oregon, USA). One fish from the three in the demonstrator group was selected at random and its movement tracked for the duration of the trial. Strike rate was quantified using raw count data, i.e. the total number of feeding strikes performed by all three demonstrators over the course of the trial. Shoal cohesion was quantified using point sampling. At 15 second intervals, giving 24 sampling instances per replicate group, we recorded the nearest neighbor distance in body lengths for each individual within each demonstrator shoal. Distance from feeder was calculated using the same approach, only here we recorded the distance in body lengths of each individual from the feeder unit.

Data were analyzed using univariate GLMs, with activity (movement in body lengths per minute by the randomly selected focal fish), strike rate (mean strike rate per individual per minute), mean nearest neighbor distance and mean distance from feeder (both in body lengths) as dependent variables. Species (*G. aculeatus* or *P. pungitius*) and feeding rate (0, 1 or 3 prey deliveries) were included as fixed factors.

Activity levels

Activity levels increased in both species as prey delivery rate increased (F_(2, 66)_=19.48, *P*<0.001). *P. pungitius* were seen to have higher activity levels than *G. aculeatus* (F_(1, 66)_=7.66, *P*=0.007), although there was no interaction between species and prey delivery rate (F_(2, 66)_=1.17, *P*=0.31, Figure S1a).

Feeding strikes

Feeding strike rates increased in both species as prey delivery rate increased (F_(2, 66)_=69.99, *P*<0.001) and differed between species (F_(1, 66)_=10.34, *P*=0.002), in interaction with species (F_(2, 66)_=2.91, P=0.061), with the *P. pungitius* exhibiting higher strike rates in the treatments where one and three prey items were delivered (Figure S1b).

Shoal cohesion

We saw a species difference, in that the mean inter-individual distance decreased significantly in the *P. pungitius* with increasing prey delivery rate across the treatments, but not in the *G. aculeatus*, for which cohesion remained consistently significantly higher than for the *P. pungitius* (species: F_(1, 66)_=35.60, *P*<0.001, treatment: F_(2, 66)_=3.72, P=0.029, interaction: F_(2, 66)_=2.58, P=0.084, Figure S1c).

Distance from feeder

Mean distance from feeder decreased significantly with increasing prey delivery rate across the treatments (F_(2, 66)_=13.97, P<0.001). We saw no species difference (F_(1, 66)_=0.06, P=0.93), or interaction effects (F_(2, 66)_=4.22, P=0.19, Figure S1d).

|  | (a) Activity |  | (b) Feeding strikes |
| --- | --- | --- | --- |
| Body lengths / min |  | Feeding strikes / min |  |
|  | (c) Cohesion |  | (d) Distance from Feeder |
| Distance (body lengths) |  | Distance (body lengths) |  |
|  | Number of prey deliveries | | |

**Figure S1.** Four measures of demonstrator behavior under different prey delivery treatments (■ *G. aculeatus*, ■ *P. Pungitius*, mean +/- 95% CI).

**S2. Demonstrator activity: pilot experiment**

We sought to manipulate demonstrator activity levels, in order to present observers with more versus less active demonstrator groups, without affecting the other behaviors (see Part 5 in main text and Figure 5 for details) that co-vary with feeder prey delivery rate. To achieve this we varied the temperature of the water in which the fish were held, placing groups of three demonstrators in chambers containing water of either 8^o^C or 18^o^C. 8^o^C was the ambient temperature in the laboratory. Fish in the 18^o^C treatment were acclimated by placing the demonstrator chamber (30cm x 15cm x 30cm, 20cm) into a water bath and increasing its temperature from 8^o^C to 18^o^C over a 72 h period. This was sufficiently long enough to avoid any shock, and no adverse effects were seen in the fish either during or after the study. Fish in the 8^o^C were also placed in the water bath (with the heater switched off) for 72 hours, and were not subjected to any temperature increase. The demonstrators were tested in demonstrator chambers measuring 30cm x 15cm x 30cm, 20cm water depth, In order to confirm the efficacy of this design we first ran pilot experiments in which 40 groups of three *G. aculeatus* and 40 groups of 3 *P. pungitius* were held at these temperatures (20 groups per temperature per species, as described above), so that their activity levels could be quantified. Each group was filmed using a camera mounted above the demonstrator chamber and we tracked the swimming rate of one of the three demonstrators, selected at random, for a 6-minute period. Six minutes was selected as this is the length of the demonstration period in the observer binary choice trials. Activity rate, converted into body lengths travelled per minute, was quantified using the manual tracking program Logger Pro (Vernier Software & Technology, Oregon, USA). Experiments were performed in March-June 2008. Activity rates were analysed using a GLM with species and temperature as fixed factors.

We found that fish were more active in the 18^o^C treatment than they were in the 8^o^C treatment (F_(1, 76)_ =43.41, P<0.001, Figure S2). There was no difference between the two species (F_(1, 76)_ =1.09, P=0.30), and no interaction effect between temperature and species (F_(1, 76)_ =0.06, P=0.81).

For comparison, the *G. aculeatus* tested in the colder and warmer water treatments here did not differ in their activity from those tested in the demonstrator behavior 1 prey and 3 prey delivery treatments ((One way ANOVAs: F_(1, 30)_= 2.45, P=0.12 and F_(1, 30)_= 0.05, P=0.82, see above and Figure S1a). The *P. pungitius* tested in warmer water here did not differ from those tested in the 3 prey delivery treatment above (F_(1, 30)_= 0.19, P=0.67), while those tested in the colder water here were slightly less active than those tested in the 1 prey delivery treatment (F_(1, 30)_= 6.19, P=0.02).

| Activity |  |
| --- | --- |
|  | Water temperature (^o^C) |

**Figure S2.** Activity (body lengths moved / minute) under low and high water temperature treatments (■ *G. aculeatus*, ■ *P. Pungitius*, mean +/- 95% CI). See main text section 3a for details.

**S3. Demonstrator strike Rate: pilot experiment**

Here we aimed to manipulate demonstrator feeding strike rates, without affecting other aspects of their behavior that co-vary with feeder prey delivery rate (see Part 5 in main text and Figure 5 for details). As prey chemical cues alone may cause fish to become more active (as increasing activity can increase encounter rates with prey) we used a non-food visual stimulus designed to elicit a feeding response upon presentation, but without any residual prey-specific chemical cues which may persist beyond the presentation of the stimulus. Both *G. aculeatus* and *P. pungitius* readily attack red objects, the result of a receiver bias probably evolved for foraging for carotanoid-rich prey (Smith et al. 2004). We exploited this by using a red laser pointer to project a dot of red light into the base of the feeder unit (see main text).

We conducted a pilot study in which 20 groups of three fish of each species were subject to either one or three 10-second exposures of the red dot, simulating the poor and rich prey delivery rates. In the three exposures group, the laser pen was switched on after 0, 2 and 4 minutes. In the one exposure group the laser pen was switched on after 2 minutes only. Experiments were performed in March-June 2008. We compared the number of total strikes made by each group, as well as the activity rate of a single, randomly selected focal fish, quantified using the same procedure as in the activity pilot experiment, described above. Both strikes rates and activity were analysed using a GLM with species and number of red dot presentations as fixed factors.

The feeding strike rate of the demonstrators was higher in the three presentations compared to one presentation treatments (F_(1, 76)_ =72.67, *P*<0.001). There was no difference between the two species (F_(1, 76)_ =0.78, *P*=0.37), and no interaction effect between stimulus exposure and species (F_(1, 76)_ =1.58, *P*=0.21, Figure S3). There were no differences in activity between the fish in the one and three exposures treatment (F_(1, 76)_ =0.53, *P*=0.47). There was no difference between the two species (F_(1, 76)_ =0.73, *P*=0.40), and no interaction effect between temperature and species (F_(1, 76)_ =0.05, P=0.83). Finally, the feeding strike rates of the fish presented with the laser stimulus were similar to those of the fish presented with (see Figure 5b), and we saw no differences in strike rates in either species between the two stimulus types (One way ANOVAs, *G. aculeatus* one and three stimulus presentations, *P. pungitius* one and three stimulus presentations: F_(1, 30)_= 0.68, P=0.42; F_(1, 30)_= 1.04, P=0.32; F_(1, 30)_= 2.25, P=0.14; F_(1, 30)_= 3.40, P=0.09).

| Feeding strikes |  |
| --- | --- |
|  | Stimulus presentations |

**Figure S3.** Feeding strikes per minute performed under one and three laser stimulus presentation treatments (■ *G. aculeatus*, ■ *P. Pungitius*, mean +/- 95% CI).

| (a) Activity | 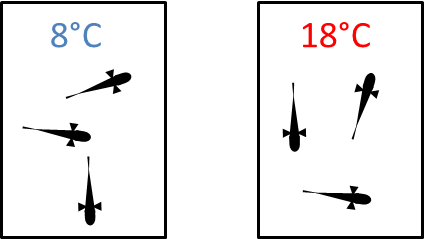 |
| --- | --- |
| (b) Feeding strikes | 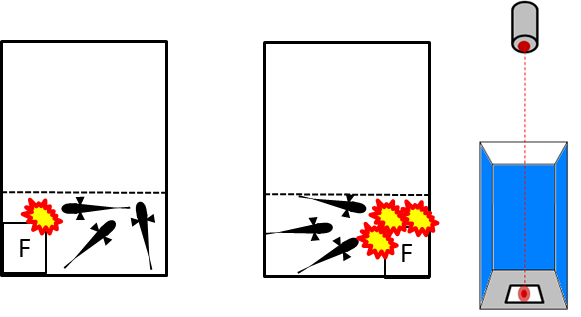 |
| (c) Cohesion | 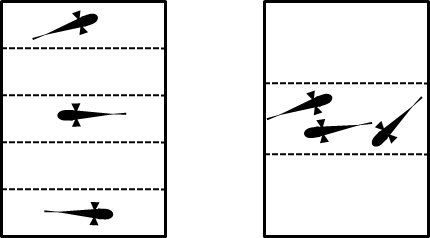 |
| (d) Distance from feeder | 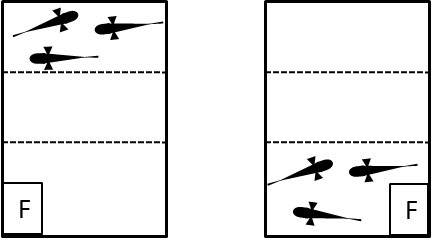 |

**Figure S4**. The modified demonstrator chambers used to present demonstrator behavior cues to the observers. In each case the chamber on the left represents the lower feeding rate demonstrator behavior and the chamber on the right the higher feeding rate demonstrator behavior (low vs high activity (a), strikes rates (b), cohesion (c) and greater versus lesser distance from feeder (d) respectively). Note that the left / right placement of these was randomized between trials). The inset figure in (b) shows the modified feeder unit (front view) in which the red lase pointer stimulus was presented. ‘F’ indicates the position of the feeder unit, where present. Not to scale. For further details see main text Section 3a.

**S4. Relative importance of demonstrator strike rate and activity level cues**

Before conducting the experiment proper we first performed a pilot experiment in order to quantify demonstrator activity and strike rate under the low temperature / three prey stimulus exposures and high temperature / one prey stimulus exposure conditions (below, fig. S5). Experiments were carried out in September 2009. We performed 12 replicates for each species and treatment group combination. Data were analyzed using separate GLMs for activity and strike rate. In each case, activity or strike rate were the dependent variables, while species and treatment combination (low temperature / three prey stimulus exposures and high temperature / one prey stimulus exposure) were included as fixed factors.

Subjects were more active under the high temperature / one prey stimulus exposure condition than they were under the low temperature / three prey stimulus exposures treatment (Treatment: F_(1, 44)_ =17.56, *P*<0.001). We saw no differences between the two species (F_(1, 44)_=1.29, *P*=0.26), nor any interaction effect between treatment and species (F_(1, 44)_=0.29, *P*=0.59). Subjects also performed more feeding strikes under the low temperature / three prey stimulus exposures condition than they were under the high temperature / one prey stimulus exposure treatment (Treatment: F_(1, 44)_ =30.95, *P*<0.001). Again we saw no differences between the two species (F_(1, 44)_=0.57, *P*=0.45), nor any interaction effect between treatment and species (F_(1, 44)_ =0.13, *P*=0.71, Figure S5).

|  | (a) Activity | |
| --- | --- | --- |
| Body lengths / min |  | |
|  | (b) Strike rate | |
| Feeding strikes/ min |  | |
|  | High temperature, 1 prey  stimulus presentation | Low temperature,3 prey stimulus presentations |

**Figure S5.** (a) Activity (body lengths moved / minute) and (b) strike rate (mean / minute) under 18^o^C water temperature, 1 prey stimulus presentation and 8^o^C water temperature, 3 prey stimulus presentation treatments (■ *G. aculeatus*, ■ *P. Pungitius*, mean +/- 95% CI).

**S5. Animated demonstrators**

Building the animations

The animations were built using *Blender* (www.blender.org), a free open source 3D content creation suite. We first built a scene, against which the animated fish moved. This consisted of a five-sided cube containing a rock and algae objects close to the back wall, which acted as unmoving size references. A white rectangle, representing the feeder unit as viewed by the observer fish, was placed on the extreme left of the scene. The scene was illuminated by three light sources, comprising a key (the main light), a fill (which lights the other side to avoid strong shadows) and a back light (which illuminates the object from the back). The key light used in this scene was a spot, set above the top left corner of the tank (Figure 7.)

The fish were created from a mesh cube that was shaped using a process referred to as box modelling. Photographs of a *P. pungitius* taken from various angles were displayed in the background and the mesh was formed to fit its shape. A mirror function was employed in order to produce a laterally symmetrical model. To create the eyes of the fish, a rounded ball was inserted at the right position, and then covered with half a sphere. The ball and sphere side vertices were joined to the adjacent vertices of the body mesh.

The pectoral fins were created by shaping a subdivided plane into a pectoral fin shape. The vertices on the side were then joined to the vertices on the body. The ventral, dorsal, and caudal fins as well as the spines were modelled from a mesh plane to allow them to be very thin. The side vertices were then joined to the vertices on the body, and all parts were linked to the main body to create one object.

Accurate articulation and realistic movements are crucial when creating animated animal stimuli for behavior experiments (Woo & Rieucau 2011). Videos of swimming sticklebacks were studied, to identify and recreate the motion involved in swimming in *P. pungitius*. Three key movements were identified: the tail bending, the pectoral fin beats, and the caudal fin waving. These movements were controlled by Blender bones, using two different types: the deformable bones that modify the mesh of the object, and the control bones that change the position of one or more of the deformable bones. A chain of eight linked vertebrae was inserted along the length of the model, and articulated using Blender’s spline function, allowing the spine to flex in a smooth but constrained curve. The caudal fin was articulated to permit 45° degrees of motion in the z axis. Sticklebacks are pectoral scullers, and the pectoral fins of the model were constructed so as to capture this movement. The waving motion of the fins was then key-framed for each bone at a time. This sequence was then repeated to cover the length of the motion pattern. Three chains of eight bones were extruded from the base of each pectoral fin, and articulated so as to allow realistic three dimensional movement. Finally, the model was colored with a counter shaded color scheme based on the coloration of non-reproductive adult *P. pungitius* collected from Melton Brook, UK. The outer sphere of the eye and the membranes of the fins were rendered with a translucent finish.

Each fish was animated with a unique movement path. The fish moved back and forth across the animated scene, and a bend of the tail was added to coincide with direction changes. Videos were rendered at 24 PNG format frames per second and each movement path ran for 150 s. These were looped to produce the 600 s trial videos, using a H.264 codec.

Pilot experiments

In a pilot study, we investigated the response of real fish to the animated stimuli. We carried out three experiments. In the first, real *P. pungitius* sticklebacks were given the opportunity to ‘shoal’ with a group of three animated fish, or to visit the goal zone adjacent to a monitor displaying an empty scene (*n*=18), with no animated fish present. In the second condition we presented a shoal of six and a shoal of two animated fish (*n*=26). In the third treatment we presented a shoal of three animated fish and a shoal of three real fish (*n*=21). To achieve this we replaced one of the monitors with a second 30x30x30cm tank. This was filled with substrate and water to the same depth as the tank housing the observer. This tank contained an opaque partition, dividing it into two sections. The smaller of these was 10 cm wide and faced the observer tank. The test subjects and live stimulus fish measured 35-40mm in length. No fish was used more than once. Since the observer was allowed to see the stimuli in real time, we modified the above testing procedure, such that following the habituation and demonstration phases, the animated fish and real fish remained visible during the five minute test phase, that is for a total of 900s. The animated fish moved independently of one another at a rate of approximately 12 body lengths per minute. The positions of the stimuli were determined randomly for each trial.

Shoaling preferences were assessed using paired sample t-tests for each treatment. The sticklebacks spent more time close to the larger shoal of animated demonstrators on both the 3 vs 0 and 6 vs 2 treatments (paired sample t-tests: *t*= 2.80, *df*=17, *P*=0.012, and *t*= 2.51, *df*=25, *P*=0.020, respectively). When presented with a choice between shoaling with three animated fish or three live fish subjects showed no preference for spending more time with either shoal (*t*= -1.74, *df*=20, *P*=0.10, Figure S6). We note that in this third treatment, there appears to be a trend biased towards shoaling with the real fish. This may be due to the fact that the real stimulus fish interacted with the movements of the observer, something the animated fish could not do. Nevertheless, this pilot work gave us confidence that our animations were sufficiently realistic so as to be able to affect the behavior of living observers. These animations were then used in the full experiment, described in the main text and Figures 7 & 8.

| Time | * * ns |
| --- | --- |

Figure S6. Pilot study investigating responses of fish to animated stimuli. Proportion of time spend in goal zone (mean +/- 95% CI). Solid bars: larger animated shoal, hatched bars: smaller animated shoal / shoal of real fish, ns: no significant difference.

**Table S1.** Details of the species and populations of sticklebacks tested in the PI-use phylogenetic comparison. *WC= wild caught, LB= lab bred, F1s, B= both (n=wild caught, n= lab bred F1s). †Location of laboratory where tests were performed. Calgary (Department of Biological Sciences, University of Calgary, Canada); Helsinki (Department of Biosciences, University of Helsinki, Finland); Mishima (National Institute of Genetics, Mishima, Japan); Salt Lake City (Department of Biology, University of Salt Lake City, Utah, USA); Seattle (Fred Hutchinson Cancer Research Centre, Seattle, USA); St Andrews (School of Biology, University of St Andrews, UK). ‡A= Artemia, BW= bloodworm, Chironomous sp. larvae, M= Mysis shrimp. Demonstrators were fed the food they received under normal feeding in the lab where they were housed. §Date that testing occurred (Month/Year).

| **Population** | **Abbrev.** | **Origin *** | **n** | **Location** † | **Prey**‡ | **When** § |
| --- | --- | --- | --- | --- | --- | --- |
| ***Gasterosteus***  *G. aculeatus* | | | | | |  |
| **Paxton benthic**, Paxton Lake,  Haida Gwaii, BC, Canada | P(b) | LB | 17 | Seattle | M | 04/2010 |
| **Paxton limnetic**, Paxton Lake, Haida Gwaii, BC, Canada | P(l) | LB | 19 | Seattle | M | 04/2010 |
| **Little Campbell**, WA, USA | LC | LB | 20 | Seattle | M | 04/2010 |
| **Lake Washington**, WA, USA | LW | LB | 20 | Seattle | M | 04/2010 |
| **Trent**, UK | Tre | WC | 40 | St Andrews | BW | Table S2 |
| **Nene**, UK | Nen | WC | 40 | St Andrews | BW | Table S2 |
| **Welland**, UK | Wel | WC | 40 | St Andrews | BW | Table S2 |
| **Great Eau**, UK | Eau | WC | 60 | St Andrews | BW | Table S2 |
| **Tees**, UK | Tee | WC | 20 | St Andrews | BW | Table S2 |
| **Ouse**, UK | Ous | WC | 20 | St Andrews | BW | Table S2 |
| **St Andrews Bay**, UK | StA | WC | 60 | St Andrews | BW | Table S2 |
| **Baltic Sea**, Uutela, Finland | Bal | WC | 15 | Helsinki | BW | 05/2011 |
| **Shiormi River**, Hokkaido, Japan | Shi | WC | 12 | Mishima | A | 05/2012 |
| **Ogaki**, Gifu, Japan | Gif | WC | 9 | Mishima | A | 05/2012 |
| **Japan Pacific**, Akkeshi Bay, Hokkaido, Japan | PJ | WC | 14 | Mishima | A | 05/2012 |
| *G. nipponicus*  **Sea of Japan**, Akkeshi Bay, Hokkaido, Japan | SoJ | WC | 13 | Mishima | A | 05/2012 |
| ***Pungitius***  *P. pungitius* | | | | | |  |
| **Church Road**, Wasilla, AK, USA | Chu | LB | 20 | Utah | BW | 12/2009 |
| **Salt River**, AB, Canada | Sal | LB | 20 | Utah | BW | 02/2010 |
| **Point McKenzie**, AK, USA | McK | B (20,20) | 40 | Utah | BW | 12/2009 |
| **Prince Edward Island**, Canada | PEI | LB | 15 | Utah | BW | 02/2010 |
| **Lough Ennell**, Ireland | LE | WC | 20 | Utah | BW | 02/2010 |
| **Trent**, UK | Tre | B (40, 12) | 52 | St Andrews | BW | Table S2 |
| **Welland**, UK | Wel | WC | 40 | St Andrews | BW | Table S2 |
| **Nene**, UK | Nen | WC | 40 | St Andrews | BW | Table S2 |
| **Great Eau**, UK | Eau | WC | 60 | St Andrews | BW | Table S2 |
| **Baltic Sea**, Uutela, Finland | Bal | B (10,10) | 20 | Helsinki | BW | 05/2011 |
| **Rytilampi**, Kuusamo, Finland | Ryt | WC | 10 | Helsinki | BW | 05/2011 |
| **Pyӧreälampi**, Kuusamo, Finland | Pyo | WC | 16 | Helsinki | BW | 05/2011 |
| **Biwase**, Hokkaido, Japan | Biw | WC | 15 | Mishima | A | 05/2012 |
| **Hyotan Pond**, Hokkaido, Japan | Hyo | WC | 7 | Mishima | A | 05/2012 |
| ***P. sinensis***  Oboro River, Hokkaido, Japan | P.s | WC | 8 | Mishima | A | 05/2012 |
| ***Culaea inconstans*** | | | | | |  |
| **Delta Marsh**, MB, Canada | Del | WC | 11 | Utah | BW | 12/2009 |
| **Birch**, AB, Canada | Bir | WC | 20 | Calgary | BW | 01/2011 |
| **Gasplant**, AB, Canada | Gas | WC | 20 | Calgary | BW | 01/2011 |
| **Teal,** AB, Canada | Tea | WC | 20 | Calgary | BW | 01/2011 |
| **Crimson**, AB, Canada | Cri | WC | 15 | Calgary | BW | 01/2011 |
| **Fiesta**, AB, Canada | Fie | WC | 18 | Calgary | BW | 01/2011 |
| ***Apeltes quadracus*** | | | | | |  |
| **West River**, CT, USA | Wes | LB | 20 | Seattle | M | 04/2010 |
| ***Spinachia spinachia*** | | | | | |  |
| **St Andrews Bay** (North Sea), UK | StA | WC | 19 | St Andrews | M | 12/2012 |

**Table S2.** Channels of origin of the *G. aculeatus* and *P. pungitius* used in comparative study. Drainage refers to the primary channel of the drainage basin, while the three channels listed within St Andrews Bay are small tidal creeks that flow directly into the North Sea. *Only predatory fishes were recorded. Predatory birds were likely visitors at all collection sites. BT= brown trout (*Salmo trutta*), C= chub (*Squalius cephalus*), EP= Eurasian perch (*Perca fluviatilis*), F= Flounder (*Platichthys flesus*), NP= northern pike (*Esox lucius*). Low= no predatory fish recorded in channel during sampling. †Date that testing occurred (Month/Year).

| **Site** | | | **Species** | **Predation regime *** | **When** † |
| --- | --- | --- | --- | --- | --- |
| **Channel** | **Abbrev.** | **Drainage** |  |  |  |
| Melton Brook | Mel | Trent | Both | Low | 04/2007 |
| River Soar | Soa | Trent | Both | EP, NP | 04/2007 |
| Nosely Brook | Nos | Welland | Both | Low | 04/2007 |
| River Welland | Wel | Welland | Both | BT, C, EP, NP | 05/2007 |
| Slade Brook | Sla | Nene | Both | Low | 05/2007 |
| River Ise | Ise | Nene | Both | BT, C, EP, NP | 05/2007 |
| Howden’s Drain | How | Great Eau | Both | Low | 11/2007 |
| Great Eau | Eau | Great Eau | Both | BT, EP, NP, F | 10/2007 |
| North Creek | Nor | Great Eau | *P. pungitius* | BT, EP, NP, F | 10/2007 |
| Donna Nook | Don | Great Eau | *G. aculeatus* | Low | 11/2007 |
| Risedale Beck | Ris | Ouse | *G. aculeatus* | BT, C, EP, NP | 08/2007 |
| Clow Beck | Clo | Tees | *G. aculeatus* | BT, C, EP, NP | 08/2007 |
| Moonzie Burn | Moo | St Andrews Bay | *G. aculeatus* | BT, F | 05/2008 |
| Kinnessburn | Kin | St Andrews Bay | *G. aculeatus* | BT, F | 03/2008 |
| Kenly Water | Ken | St Andrews Bay | *G. aculeatus* | BT, F | 05/2008 |
